# An hiPSC-derived multi-lineage lung model exhibiting proximal-distal epithelial features for modeling pulmonary fibrosis

**DOI:** 10.3389/fcell.2026.1914941

**Published:** 2026-08-25

**Authors:** Chenfeng Hua, Chaojie Qu, Liu Yang, Wenfei Zhao, Junwei Zhao, Yongliang Jia, Chengjie Ma, Pingping Shang, Xiang Li, Fuwei Xie, Cong Nie

**Affiliations:** 1 Key Laboratory of Tobacco Chemistry, Zhengzhou Tobacco Research Institute of CNTC, Zhengzhou, China; 2 Beijing Life Science Academy, Beijing, China; 3 Department of Respiratory and Critical Care Medicine, The Fifth Affiliated Hospital of Zhengzhou University, Zhengzhou, China; 4 Henan Institute of Medical and Pharmaceutical Sciences, Zhengzhou University, Zhengzhou, China

**Keywords:** epithelial-mesenchymal co-development, hiPSCs, idiopathic pulmonary fibrosis, lung organoid, TGF-β1

## Abstract

**Background:**

Idiopathic pulmonary fibrosis (IPF) is a progressive and irreversible interstitial lung disease with limited therapeutic options. Existing hiPSC-derived lung organoid models are largely restricted to single epithelial lineages and cannot endogenously integrate multiple pulmonary cell types, limiting mechanistic studies and drug screening.

**Methods:**

We established a staged directed differentiation protocol to sequentially differentiate hiPSCs into definitive endoderm, anterior foregut endoderm, and lung progenitor cells, ultimately generating a multi-lineage lung model on Transwell inserts. The model was characterized by bright-field microscopy, hematoxylin-eosin (H&E) staining, scanning and transmission electron microscopy, immunofluorescence, quantitative real-time PCR (qRT-PCR), enzyme-linked immunosorbent assay (ELISA), and single-cell RNA sequencing. Fibrosis-like phenotypes were induced by transforming growth factor-beta 1 (TGF-β1) stimulation. Transcriptomic similarity to human IPF was assessed by RNA sequencing (RNA-seq) combined with bidirectional gene set enrichment analysis (GSEA), and drug responsiveness was validated with pirfenidone.

**Results:**

Without genetic editing or exogenous cell supplementation, the model endogenously generated proximal duct-like epithelium, distal alveolar epithelium, fibroblasts, endothelial cells, and CD68^+^ macrophage-like cells confirmed by immunofluorescence. Single-cell sequencing identified 13 transcriptional subclusters and revealed epithelial-mesenchymal co-development. TGF-β1 stimulation induced epithelial barrier disruption, ferroptosis-like mitochondrial ultrastructural alterations, extracellular matrix (ECM) remodeling, and aberrant secretion of multiple IPF-associated biomarkers. Transcriptomic analysis showed that upregulated genes in the fibrosis model group were significantly enriched in ECM organization, cell adhesion, and the PI3K-Akt pathway. Protein-protein interaction (PPI) network analysis identified *COL1A1*, *FN1*, and integrin family members as central hubs. Bidirectional GSEA validation confirmed that the transcriptomic signature of the model was highly similar to human IPF, with differentially expressed genes significantly enriched in three independent IPF cohorts. Pirfenidone reversed TGF-β1-induced ECM deposition and myofibroblast activation, and partially restored type II alveolar epithelial (AT2) cell marker expression.

**Conclusion:**

We successfully established a humanized multi-lineage lung model that captures epithelial-mesenchymal co-development *in vitro*. Its responsiveness to TGF-β1 stimulation and sensitivity to pirfenidone highlight its potential for antifibrotic drug screening. This platform offers a humanized tool with the potential to be standardized for investigating cell fate determination during early lung development and the pathogenesis of IPF.

## 1 Introduction

Idiopathic pulmonary fibrosis (IPF) is a chronic, progressive, and irreversible interstitial lung disease with a median survival of only 2.5–3.5 years. The disease carries high morbidity and mortality, severely compromising patients’ quality of life and posing a significant threat to their survival (Society, 2000; Benegas Urteaga et al., 2022). To date, three antifibrotic agents—pirfenidone (PFD), nintedanib, and nerandomilast—have been approved for IPF treatment in multiple countries, including the United States, China, and Japan. However, none of these agents reverses or completely halts disease progression, and all are frequently associated with notable adverse effects (Sgalla et al., 2016; Lancaster et al., 2019; Behr et al., 2020). Therefore, the development of more effective therapeutic strategies remains a core challenge in the field. Overcoming this bottleneck requires a deeper understanding of IPF pathogenesis and the establishment of humanized models capable of reliably evaluating potential antifibrotic agents.

The hallmark histopathological feature of IPF is usual interstitial pneumonia (UIP), characterized by excessive deposition of extracellular matrix (ECM) components such as collagens and fibronectin, aberrant proliferation of fibroblasts and their differentiation into myofibroblasts, and eventual destruction of alveolar architecture leading to impaired gas exchange (Tanner et al., 2023; Xiao et al., 2024; Gao et al., 2025). Substantial evidence indicates that IPF pathogenesis involves complex crosstalk among multiple cell types. Repeated injury and aberrant repair of alveolar epithelial cells, particularly type II alveolar epithelial (AT2) cells, are considered the initiating event of IPF (Parimon et al., 2020). Injured epithelial cells secrete factors such as transforming growth factor-β (TGF-β) and platelet-derived growth factor (PDGF), which activate fibroblasts and promote their differentiation into α-smooth muscle actin (α-SMA)-expressing myofibroblasts, a process termed fibroblast-to-myofibroblast transition (FMT), which leads to massive ECM production (Hult et al., 2022; Liu et al., 2023). In addition, epithelial-mesenchymal transition (EMT) has been recognized as another distinct cellular mechanism contributing to fibrosis (Salton et al., 2020; Ishida et al., 2023). TGF-β serves as a central driver of this network, promoting fibroblast activation and ECM deposition (Su et al., 2020; Lu et al., 2023; Cohen et al., 2024). Meanwhile, immune cells (e.g., macrophages) and stromal cells (e.g., endothelial cells) participate in disease progression by establishing a profibrotic microenvironment (Wynn and Vannella, 2016). Given the complexity of these intercellular interactions, recent single-cell transcriptomic studies have further unveiled highly heterogeneous cell subpopulations and their aberrant communication networks in IPF lung tissues, underscoring the urgent need to recapitulate such multicellular pathological ecosystems *in vitro* (Xu et al., 2024; Reyfman et al., 2019; Adams et al., 2020; Jiang et al., 2025). However, such models remain lacking, limiting mechanistic studies and drug screening.

These limitations are further compounded by conventional research models. Two-dimensional cell cultures fail to recapitulate tissue architecture and cell-cell communication (Pampaloni et al., 2007), while animal models, due to species differences, exhibit fundamental discrepancies in cellular composition, molecular regulation, and drug responsiveness, making them inadequate for faithfully reflecting the complex pathological features of human IPF (Kolanko et al., 2024). The advent of human induced pluripotent stem cells (hiPSCs) and organoid technology has opened new avenues for constructing humanized lung models (Lee et al., 2021). Several studies have differentiated hiPSCs into alveolar or airway epithelial cells and generated lung organoids, partially reconstituting cellular interactions by exogenously adding fibroblasts or immune cells (Yamamoto et al., 2020; Pezet et al., 2026). However, these advanced models still face two major bottlenecks. First, epithelial lineage completeness remains limited: most protocols yield only a single epithelial lineage—either alveolar or airway—and do not endogenously generate both proximal and distal epithelial populations within a single system (McCauley et al., 2017; Miller et al., 2019). Second, stromal and immune cells rely on exogenous integration: key mesenchymal cells (e.g., fibroblasts) and immune cells typically require exogenous supplementation at later culture stages, which differs from the self-assembled ecological microenvironment formed by multicellular synergy *in vivo* (Barkauskas et al., 2017; Hein et al., 2022). Accordingly, there is an urgent need to construct a humanized model that endogenously integrates multiple pulmonary cell types—including epithelial, fibroblast, and endothelial components—to support mechanistic studies and drug screening for IPF.

To address these bottlenecks, a humanized multi-lineage lung model was established that endogenously generates proximal duct-like epithelium, distal alveolar epithelium, fibroblasts, endothelial cells and macrophage-like cells within a single system. Through multi-dimensional characterization encompassing histology, ultrastructure, and single-cell transcriptomics, the model was shown to capture epithelial-mesenchymal co-development and to possess the structural basis for multicellular crosstalk. Upon TGF-β1 stimulation, fibrosis-like phenotypes were induced in this system, including epithelial barrier damage, ferroptosis-like ultrastructural changes, ECM remodeling, and aberrant secretion of multiple IPF-associated biomarkers. Furthermore, through a systematic bidirectional gene set enrichment analysis (GSEA) strategy, the transcriptomic signature of the fibrotic model was verified to be highly consistent with three independent human IPF cohorts, and these pathological changes were effectively reversed by the clinical drug PFD. This study provides a humanized multicellular model platform with potential for antifibrotic drug evaluation and investigation of multicellular interaction mechanisms in IPF.

## 2 Materials and methods

### 2.1 Cell culture and differentiation

hiPSCs (iCell-DRY0100, iCell Bioscience, China) were maintained under feeder-free conditions in mTeSR™1 medium (85,851, STEMCELL Technologies, Canada) on 6 cm dishes (430,166, Corning, United States) pre-coated with Matrigel® basement membrane matrix (354,277, Corning, United States). At approximately 80% confluence, cells were rinsed with DPBS (14190144, Gibco, United States), dissociated with Accutase (A1110501, Gibco, United States), and pipetted gently to obtain a single-cell suspension. After Trypan Blue staining (15250061, Gibco, United States), viable cells were counted and seeded at a density of 1.0 × 10^5^ cells/mL onto the upper chamber of 24-well Transwell inserts with a pore size of 0.4 μm (3470, Corning, United States). Following cell attachment, the directed differentiation program was initiated. All culture steps were performed at 37 °C in a humidified atmosphere with 5% CO_2_.

To generate the multi-lineage lung model, a staged directed differentiation protocol was used. Briefly, hiPSCs were first differentiated into definitive endoderm (DE) using Activin A, rhBMP4, Y-27632, and CHIR99021 for 4 days. DE cells were then treated with Noggin and SB431542 for 3 days to induce anterior foregut endoderm (AFE) differentiation. Subsequently, lung progenitor (LP) differentiation was performed in medium containing rhFGF-7, rhFGF-10, rhBMP-4, all-trans retinoic acid (ATRA), CHIR99021, rhEGF, and rhFGF-2 for 10 days. Finally, the medium was switched to differentiation medium containing CHIR99021, rhFGF-7, rhFGF-10, dexamethasone, 8-Br-cAMP, IBMX, and ITS, and differentiation was continued for another 10 days to obtain the multi-lineage lung model. The basal medium for all differentiation stages was DMEM/F-12 (with GlutaMAX) supplemented with B-27, N-2, penicillin-streptomycin, fetal bovine serum, monothioglycerol, and L-ascorbic acid. Detailed working concentrations, suppliers, and catalog numbers are provided in Supplementary Material S1, Supplementary Tables S1–S6.

### 2.2 TGF-β1-induced fibrosis model

To induce fibrotic responses, fully differentiated multi-lineage lung models (day 27) were treated with 10 ng/mL recombinant human TGF-β1 (240-B, R&D Systems, United States) for 72 h. Control cultures received basal medium without stimulating factors throughout the experiment. After treatment, the models and culture supernatants were collected for qRT-PCR analysis of fibrosis-related marker expression, ELISA, transcriptome sequencing (RNA-seq), and single-cell RNA sequencing (scRNA-seq).

### 2.3 Pirfenidone intervention experiments

To evaluate drug responsiveness, fully differentiated multi-lineage lung models (day 27) were co-treated with 10 ng/mL TGF-β1 with or without 1 mg/mL pirfenidone (PFD) (P129335, Aladdin, China) for 72 h. PFD was prepared freshly, dissolved in alveolar fluid to generate a 10 mg/mL stock solution, and diluted to the working concentration with alveolar fluid immediately before use. Experimental groups included: medium control, PFD alone, TGF-β1 treatment, and TGF-β1+PFD co-treatment. After treatment, the models and culture supernatants were collected for qRT-PCR and immunofluorescence analysis.

### 2.4 Gene expression analysis

Total RNA was extracted using a Total RNA Isolation Kit (082,001, Beibei Biotechnology, China). cDNA was synthesized using the PrimeScript™ RT Reagent Kit with gDNA Eraser (RR047A, Takara Tomy, Japan), and quantitative real-time PCR (qRT-PCR) was performed using TB Green® Premix Ex Taq™ II (RR820A, Takara Tomy, Japan) on a LightCycler® 96 Instrument (LightCycler 96, Roche, Switzerland). β-Actin served as the internal reference, and relative gene expression was calculated using the 2^−ΔΔCt^ method. Primer sequences are listed in Supplementary Material S1, Supplementary Table S7.

### 2.5 Immunofluorescence staining and quantitative image analysis

For cytospin preparation, cells from day-27 models were dissociated using Accutase, pelleted by centrifugation at 300 *g* for 5 min, resuspended in PBS, dropped onto poly-L-lysine-coated slides, and allowed to settle by gravity for 20 min. After removing the supernatant, the slides were air-dried and subjected to fixation, permeabilization, and blocking as described below.

For paraffin-embedded sections, the collected models were fixed with fixative solution overnight at 4 °C, washed with PBS, and entrusted to Wuhan Servicebio Technology Co., Ltd. For tissue processing, paraffin embedding, sectioning (4 μm thickness), and immunofluorescence staining. The staining procedure included deparaffinization, rehydration, antigen retrieval by heating in sodium citrate buffer (10 mM, pH 6.0) at 95 °C for 15 min, and blocking with blocking buffer.

For both cytospin preparations and paraffin sections, samples were fixed (for cytospin samples only; sections were fixed during tissue processing), rinsed with washing solution (P0106, Beyotime, China), permeabilized with permeabilization buffer (P0097, Beyotime, China) for 10 min (for cytospin samples only), and blocked with blocking buffer (A2153, Beyotime, China) for 2–6 h at room temperature.

Samples were then incubated with primary antibodies overnight at 4 °C. After washing, they were exposed species-matched Alexa Fluor-conjugated secondary antibodies were applied. Nuclei were counterstained with DAPI (75,004, STEMCELL, Canada). Images were acquired using a fluorescence microscope (DMi8, Leica, Germany), and fluorescence intensity was quantified using ImageJ (1.46r) software. Results are presented as the ratio of the total fluorescence area of the target protein to the DAPI-positive area. Antibodies and dilution ratios are listed in Supplementary Material S1, Supplementary Table S8.

### 2.6 Barrier function and cell viability assessment

Barrier function was evaluated by ZO-1 immunofluorescence staining as described in Section 2.5. Cell viability was assessed using a CCK-8 Kit (CK-04, Dojindo Laboratories, Japan) according to the manufacturer’s instructions. Cell membrane integrity was further evaluated by measuring lactate dehydrogenase (LDH) release into the culture supernatant using a commercial LDH cytotoxicity assay kit (CK12, Dojindo Laboratories, Japan), following the manufacturer’s protocol.

### 2.7 ELISA

The concentrations of profibrotic factors and inflammatory mediators in culture supernatants were measured using commercial enzyme-linked immunosorbent assay (ELISA) kits strictly according to the manufacturers’ protocols. The list of kits used is provided in Supplementary Material S1, Supplementary Table S9.

### 2.8 Histological analysis

The collected multi-lineage lung models were fixed with fixative solution overnight at 4 °C. After washing with PBS, samples were entrusted to Wuhan Servicebio Technology Co., Ltd. For subsequent tissue processing, paraffin embedding, sectioning (4 μm thickness), and hematoxylin-eosin (H&E) staining. Whole-section digital images were acquired using a digital slide scanner.

### 2.9 Scanning electron microscopy

Multi-lineage lung models were fixed with scanning electron microscopy fixative (P1116, Solarbio, China) overnight at 4 °C, washed with PBS, and processed by the Shiyanjia platform for post-fixation with 1% osmium tetroxide, gradient ethanol dehydration, critical-point drying, sputter-coating with gold, and observation/image acquisition via scanning electron microscopy (SEM).

### 2.10 Transmission electron microscopy

Multi-lineage lung tissue samples were fixed with electron microscopy fixative (in 0.1 M phosphate buffer, pH 7.4) overnight at 4 °C, washed with buffer, and processed by the Shiyanjia platform for post-fixation with 1% osmium tetroxide, gradient ethanol dehydration, epoxy resin embedding, and ultrathin sectioning (60–80 nm) with double staining of uranyl acetate and lead citrate, followed by observation and image acquisition via transmission electron microscopy (TEM). Special attention was paid to epithelial tight junctions, microvilli, cilia, and mitochondrial ultrastructure.

### 2.11 Single-cell RNA sequencing analysis

Multi-lineage lung models at day 27 were dissociated into single-cell suspensions. After Trypan Blue staining confirming cell viability (>85%), samples were entrusted to BGI for single-cell capture and library construction using the 10× Genomics platform, followed by sequencing on the DNBSEQ platform. Quality control, data normalization, dimensionality reduction, clustering, and initial cell type annotation were performed by BGI on their Dr. Tom online platform. In this study, pre-analyzed data exported from the Dr. Tom platform, including annotated cell subclusters and gene expression matrices, were used for subsequent visualization. UMAP plots, cell proportion bar charts, marker gene heatmaps/dot plots, and pseudotime trajectory plots were generated using R software packages (Seurat, Monocle 3, Slingshot, etc.) based on the analysis results derived from the Dr. Tom platform.

### 2.12 Transcriptome sequencing analysis

Transcriptome sequencing was performed on the DNBSEQ platform by BGI. Initial data processing (including quality control, read quantification, and data normalization) was performed by BGI, which provided the cleaned normalized gene expression matrix for downstream analyses. All downstream bioinformatics analyses and interpretation were performed independently by the research group. Differential expression analysis was performed using the DESeq2 R package with a negative binomial generalized linear model. Genes with an adjusted p-value (false discovery rate, FDR) < 0.05 and an absolute log_2_ fold change >1 were defined as significantly differentially expressed genes. Data visualization (including principal component analysis (PCA) plots, volcano plots, and heatmaps) was generated using R packages (ggplot2 and related visualization tools). Gene Ontology (GO) and Kyoto Encyclopedia of Genes and Genomes (KEGG) pathway enrichment analyses were performed using the clusterProfiler R package, and results were visualized via the Bioinformatics online platform (https://www.bioinformatics.com.cn). Protein-protein interaction (PPI) networks were constructed using the STRING database online platform (https://string-db.org), and the results were imported into Cytoscape (v3.10.4) for network visualization and key gene identification. Network topology parameters (including clustering coefficients and network density) were calculated using the NetworkAnalyzer tool in Cytoscape.

### 2.13 Bidirectional GSEA validation with public datasets

To validate the transcriptomic similarity between the TGF-β1-induced fibrosis group and human IPF, normalized expression matrices for three independent IPF datasets (GSE17978, GSE47460, GSE213001) were downloaded from the Gene Expression Omnibus (GEO) database. The bidirectional gene set enrichment analysis (GSEA) validation strategy was as follows: (1) IPF-associated upregulated genes from the three GEO datasets were used as the target gene set to evaluate their enrichment in the model group’s ranked gene list; and (2) upregulated genes from the model group were used as the target gene set to evaluate their enrichment in the three independent GEO datasets’ ranked gene lists. In parallel, GSEA was performed on the model group’s entire gene list against standard pathway sets (Hallmark and KEGG pathways) from the Molecular Signatures Database (MSigDB, v2023.1). All GSEA were performed using the clusterProfiler R package.

### 2.14 Statistical analysis

All experiments were independently repeated at least three times, and data are presented as mean ± standard error of the mean. The Shapiro-Wilk test was used to verify normality, and Levene’s test was used to assess homogeneity of variance for normally distributed data. For multiple-group comparisons, one-way ANOVA followed by Tukey’s HSD *post hoc* test was applied when variances were homogeneous, and Welch’s ANOVA followed by Games-Howell *post hoc* test was applied when variances were unequal. For two-group comparisons, Student’s unpaired t-test was used for normally distributed data with equal variances, Welch’s t-test was applied when variances were unequal, and the Mann-Whitney U test was used for non-normally distributed data. All tests were two-sided, and *p* < 0.05 was considered statistically significant. Statistical analyses were performed using SPSS 17.0 software (SPSS Inc., Chicago, IL, United States). Graphs were generated using GraphPad Prism 8.0 (GraphPad Software, La Jolla, CA, United States) and R software (version 4.0 or later).

## 3 Results

### 3.1 Directed differentiation of hiPSCs into functional lung progenitor cells

**FIGURE 1:**
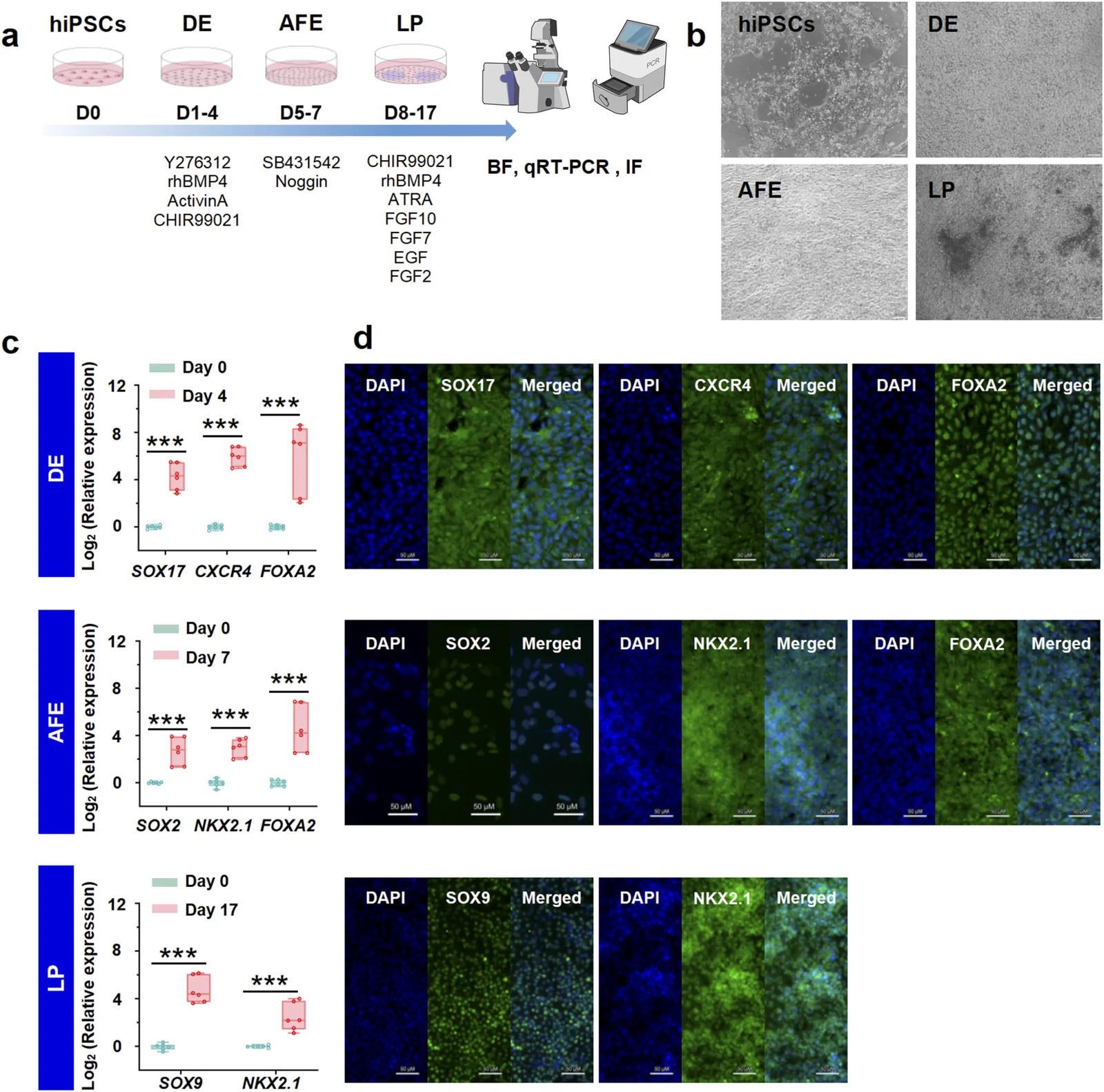
Efficient differentiation of hiPSCs into functional lung progenitor cells. **(a)** Schematic of the multi-stage directed differentiation protocol from hiPSCs to lung progenitor cells. DE, definitive endoderm; AFE, anterior foregut endoderm; LP, lung progenitors. **(b)** Representative bright-field images at each differentiation stage. Scale bars: 100 μm. **(c)** qRT-PCR analysis of relative expression (log2 scale) of key marker genes at each stage. (i) Definitive endoderm markers SOX17, CXCR4, and FOXA2; (ii) anterior foregut endoderm markers SOX2, NKX2.1, and FOXA2; (iii) lung progenitor markers SOX9 and NKX2.1. Statistical significance was determined by Welch’s t-test (each time point vs. day 0), except for NKX2.1 at day 7, which was analyzed using Student’s unpaired t-test. ***p < 0.001. **(d)** Representative immunofluorescence images of marker proteins at each differentiation stage. Scale bars: 50 μm.

To construct a humanized model containing multiple pulmonary cell types *in vitro*, we established a multi-stage directed differentiation protocol to differentiate hiPSCs stepwise into lung progenitor cells (Figure 1a). Bright-field microscopy revealed progressive morphological changes from uniform hiPSC colonies to cell populations with distinct epithelial-like features (Figure 1b).

qRT-PCR analysis confirmed the sequential activation of stage-specific markers during the differentiation process (Figure 1c). Compared with day 0, endoderm markers *SOX17*, *CXCR4*, and *FOXA2* were all significantly upregulated at the definitive endoderm (DE) stage (day 4). At the anterior foregut endoderm stage (AFE) (day 7), the foregut marker *SOX2* and the lung fate determinant *NKX2.1* were successfully induced, while *FOXA2* maintained high expression. At the lung progenitor (LP) stage (day 17), the progenitor marker *SOX9* was significantly upregulated, and *NKX2.1* remained highly expressed. These results verified key molecular events in lung lineage specification at the transcriptional level.

Immunofluorescence staining further confirmed the differentiation trajectory at the protein level (Figure 1d). Cells positive for SOX17, CXCR4, and FOXA2 were detected at the DE stage; cells positive for SOX2, NKX2.1, and FOXA2 at the AFE stage; and cells double-positive for NKX2.1 and SOX9 at the LP stage, strongly confirming the identity of lung progenitor cells.

### 3.2 Morphological, ultrastructural, and stromal cell characterization of the multi-lineage lung model

**FIGURE 2:**
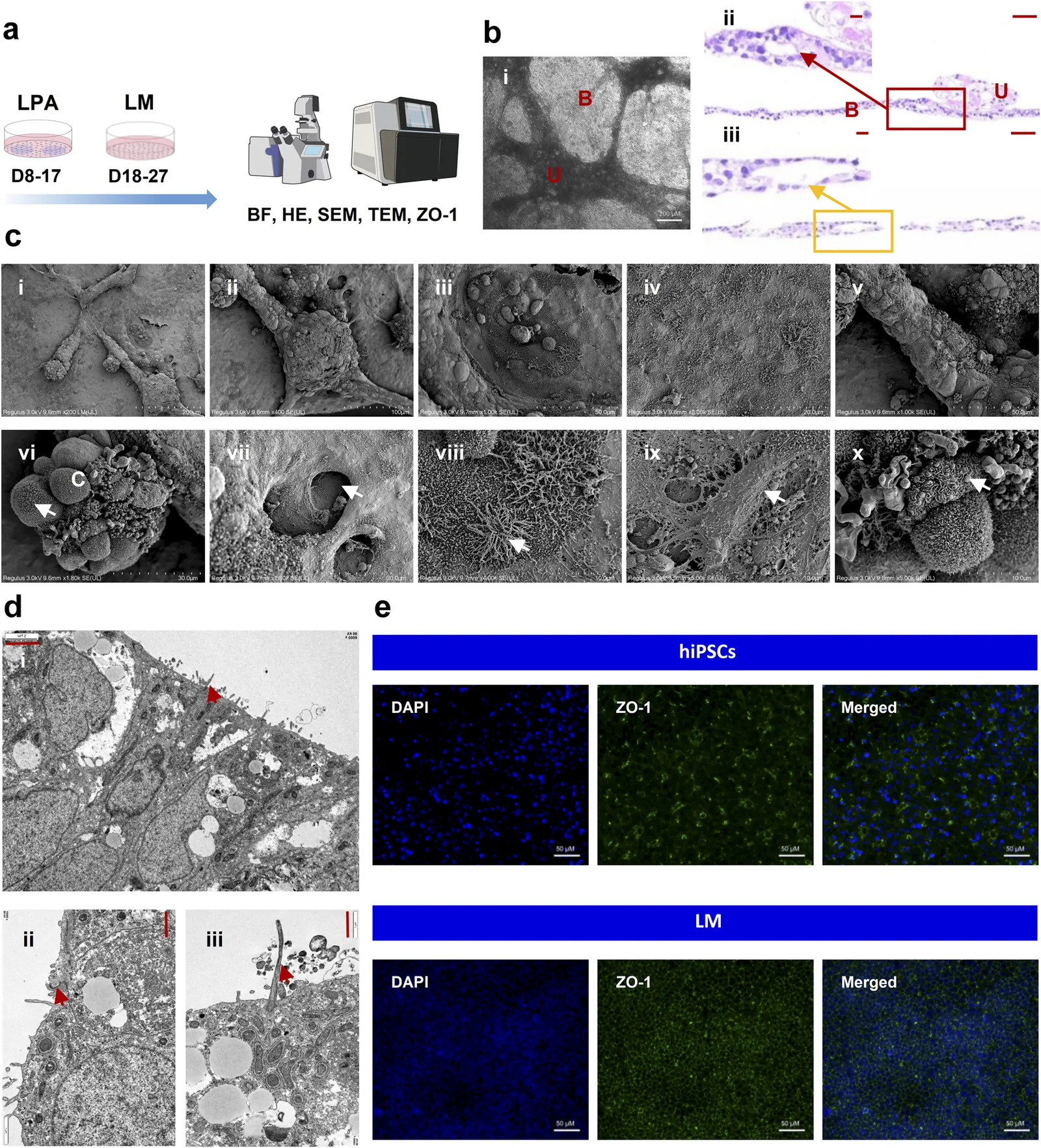
Morphological, ultrastructural, and barrier function characterization of the multi-lineage lung model (LM). **(a)** Schematic workflow of differentiation from lung progenitors to the multi-lineage model and characterization. **(b)** Bright-field images and H&E-stained longitudinal sections of day-27 models. Red arrow: duct-like lumina; yellow arrow: alveolus-like cavities; B: basal layer; U: upper layer. Bright-field scale bar: 200 μm; H&E scale bars: left 50 μm, right 10 μm. **(c)** Scanning electron microscopy (SEM) images showing surface topography and cellular diversity. (i-iv) Overall surface morphology of the model; (v) branched ridges and undulating textures; (vi) surface spherical cell clusters and microvilli-like protrusions; (vii) pore-like cavities; (viii) cells with ciliary structures; (ix) spindle-shaped cells and interwoven fibrous network; (x) cells with surface undulating membrane ruffling characteristics. Arrows: microvilli-like protrusions (vi), pore-like cavities (vii), cilia (viii), fibroblasts (ix), membrane ruffling (x); C: spherical cell clusters. Scale bars: 200, 100, 50, 20, 50, 30, 30, 10, 10, and 10 μm, respectively. **(d)** Transmission electron microscopy (TEM) images. Arrows: microvilli (i), tight junctions, cilia (iii); scale bars: 2, 1, and 1 μm, respectively. **(e)** ZO-1 immunofluorescence staining. Scale bar: 50 μm.

Lung progenitor cells at day 17 were further differentiated to day 27 to obtain the multi-lineage lung model, which was then subjected to multi-dimensional structural and functional characterization (Figure 2a).

At the macroscopic and histological levels, the submerged-culture model displayed characteristic biphasic structures under bright field: a compact, cell-dense basal layer and a loose, reticular membrane-like upper layer, with occasional round or oval contours enclosed by the reticular network (Figure 2b). H&E-stained longitudinal sections further revealed laminar architecture: the basal layer exhibited pseudostratified epithelial with duct-like lumina surrounded by cuboidal or columnar epithelium and alveolus-like cavities lined by flattened cells; the upper layer consisted of a loose eosinophilic stroma with scattered nuclei and vacuole-like structures (Figure 2b).

At the ultrastructural level, scanning electron microscopy (SEM) revealed a rough, uneven surface (Figure 2c i-iv) with branched ridges, undulating textures (Figures 2c ii,v), and attached spherical cell clusters (Figure 2c ii, vi), constituting a complex three-dimensional architecture. Well-demarcated pore-like cavities resembling alveolar spaces were also observed on the surface (Figure 2c vii). Regarding cellular composition, the surface contained spherical cells densely covered with short microvilli-like protrusions (Figure 2c i, ii, vi, x) and surrounded by secretory granules, resembling AT2 cell morphology. Flattened, smooth-surfaced areas were also present, suggestive of transitional alveolar epithelial cells (Figure 2c iii, iv, vii). Occasional cells with typical cilia were noted (Figure 2c iii, viii), suggesting endogenous proximal airway differentiation. For stromal components, spindle-shaped cells with elongated processes were observed in the upper layer and interepithelial spaces (Figure 2c iii, v, ix), surrounded by an interwoven fibrous network consistent with ECM components such as collagen fibers, suggesting active matrix secretion and assembly. Notably, cells with surface undulating membrane ruffling were observed in ridge regions (Figures 2c vi,x)—a feature potentially suggestive of macrophages, although this observation alone cannot confirm their identity—providing ultrastructural morphological clues for the possible presence of immune cell.

Transmission electron microscopy (TEM) further revealed epithelial differentiation and barrier structures at the subcellular level. Epithelial regions exhibited pseudostratified columnar morphology with nuclei at varying heights (Figure 2d i); typical tight junctions were observed between adjacent epithelial cells (Figure 2d ii); microvilli were distributed on the apical surface, and a few cells displayed apical cilia (Figure 2d iii). ZO-1 immunofluorescence staining further demonstrated continuous linear distribution of tight junction proteins along cell borders in day-27 models, confirming the formation of an intact tight junction network and corroborating the TEM observations (Figure 2e).

### 3.3 Single-cell transcriptomic sequencing reveals cellular heterogeneity and proximal-distal epithelial differentiation in the model

**FIGURE 3:**
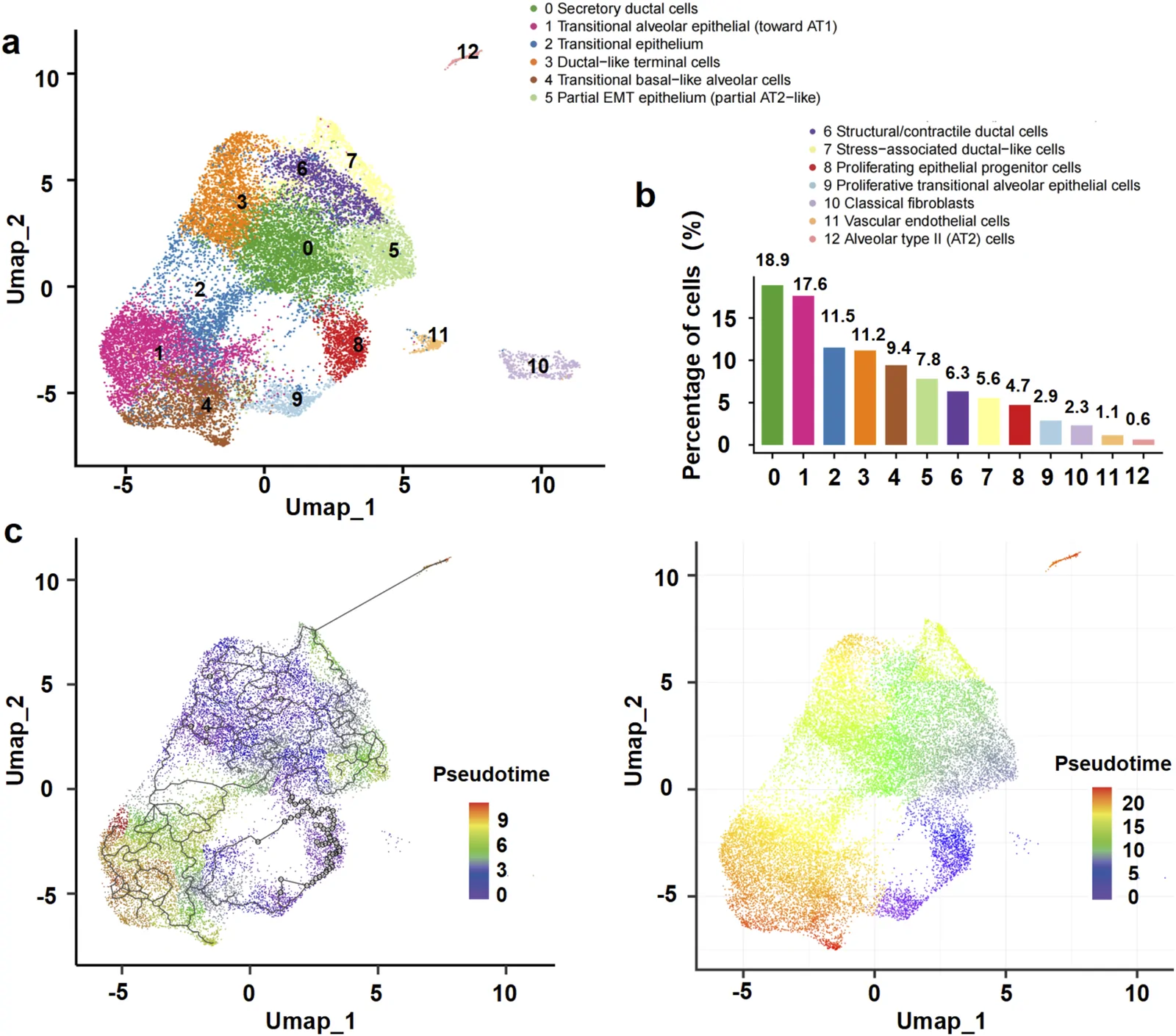
Single-cell transcriptomic sequencing reveals cellular heterogeneity and proximal-distal epithelial differentiation in the model. **(a)** UMAP dimensionality reduction plot with 13 cell subclusters (clusters 0–12) annotated in distinct colors. **(b)** Bar chart showing the proportion of each cell subcluster. **(c)** Pseudotime analysis. Left: Monocle 3 pseudotime trajectory; Right: Slingshot pseudotime trajectory.

To systematically dissect the cellular composition at the transcriptomic level, we performed single-cell RNA sequencing on the day-27 models. After stringent quality control, 21,132 high-quality single-cell transcriptomes were obtained, and unsupervised clustering identified 13 transcriptionally distinct cell subclusters (Figure 3a).

Based on marker genes expression and functional enrichment analysis (Table 1; Supplementary Materials S2, S3; Supplementary Document S1), we annotated the 13 subclusters, which encompassed proximal ductal epithelium (*KRT7* ^+^/*KRT19* ^+^), distal alveolar epithelium (*SFTPC* ^+^/*AGER* ^+^), fibroblasts (*COL1A1* ^+^), endothelial cells (*PECAM1* ^+^), and other cell types. Subcluster names, proportions, and core marker genes are summarized in Table 1. Cell proportion analysis revealed that ductal/ductal-like epithelium accounted the highest proportion (clusters 0, 3, 6, and 7; ∼41.9%), followed by distal alveolar lineages (clusters 1, 4, and 12; ∼27.7%), with fibroblasts (cluster 10, 2.31%), endothelial cells (cluster 11, 1.15%), and proliferative epithelial cells (clusters 8–9, 7.6%) collectively accounting for approximately 11% (Figure 3b).

**TABLE 1:** Cell subclusters identified by single-cell sequencing, proportions, and core marker genes.

Cluster	Name	Cell count	Proportion (%)	Core marker genes
0	Secretory ductal cells	3,988	18.87	*KRT7, KRT19, WFDC2, EPCAM*
1	Transitional alveolar epithelial (toward AT1)	3,724	17.62	*MACC1, FGD4, RAPGEF2, AGER*
2	Transitional epithelium	2,431	11.5	*CDH1, FGD4, RAPGEF2*
3	Ductal-like terminal cells	2,359	11.16	*KRT7, HOPX, CALD1*
4	Transitional basal-like alveolar cells	1,993	9.43	*TP63, AGER, RAPGEF2, FGD4*
5	Partial EMT epithelium (partial AT2-like)	1,654	7.83	*EPCAM, VIM, COL1A1, FN1*
6	Structural/contractile ductal cells	1,337	6.33	*KRT7, KRT19, MYL9*
7	Stress-associated ductal-like cells	1,174	5.56	*DDIT4, HIF1A, NUPR1, PHLDA2*
8	Proliferating epithelial progenitor cells	998	4.72	*MKI67, TOP2A, CDK1*
9	Proliferative transitional alveolar epithelial cells	608	2.88	*MKI67, MACC1, RAPGEF2, AGER*
10	Classical fibroblasts	489	2.31	VIM, COL1A1, DCN, PDGFRA
11	Vascular endothelial cells	242	1.15	PECAM1, VWF, CDH5
12	Alveolar type II (AT2) cells	135	0.64	NKX2-1, NAPSA, SFTPB, SFTPC

The proximal epithelial population was predominantly composed of ductal-like cell types with high expression of *KRT7*, *KRT19*, and other markers. However, no independent subclusters of mature ciliated cells (*FOXJ1* ^+^), secretory club cells (*SCGB1A1* ^+^), or basal cells with a mature transcriptional program were detected. Although scattered *KRT5* ^+^ or *TP63* ^+^ cells were observed within ductal and transitional populations, they did not co-express the full repertoire of basal markers (e.g., NGFR, ITGA6) or form a distinct cluster. This suggests that proximal epithelial differentiation in the model was primarily at the ductal-like stage and had not further specialized into basal-ciliated-club lineages of the proximal airway. The distal epithelium encompassed alveolar cells transitioning toward AT1 and a small subset of genuine AT2 cells (*SFTPC* ^+^). Pseudotime analysis further revealed continuous differentiation trajectories from progenitor cells toward both proximal ductal epithelium and distal alveolar epithelium, with consistent results obtained by Monocle 3 and Slingshot (Figure 3c). In single-cell sequencing, cluster 5 (partial EMT epithelium, partial AT2-like) co-expressed epithelial (EPCAM) and mesenchymal (COL1A1, FN1) markers, and pseudotime analysis suggested that it may represent a transitional cell state during normal differentiation. GO enrichment analysis of cluster 5 under unstimulated conditions showed enrichment in mitochondrial energy metabolism and ribosome biogenesis, with no enrichment of EMT- or inflammation-related pathways (Supplementary Material S3 and Supplementary Document S1).

No independent immune cell transcriptional subclusters were captured in this scRNA-seq analysis, which is likely attributable to the extremely low abundance of such cells and the detection sensitivity limitations inherent to single-cell sequencing technology. This single-cell atlas clearly defined the major cellular composition of the model (proximal/distal epithelial lineages, stromal cells, and endothelial components) at the transcriptomic level, which was highly consistent with morphological observations.

To validate the presence of specific cell populations at the protein level, we performed immunofluorescence staining on paraffin-embedded sections from the day-27 models. Pro-SPC^+^ cells were detected, supporting the presence of alveolar type II-like cells within the distal epithelial population (Supplementary Material S4). In addition, immunofluorescence staining on dissociated cells (cytospin preparation) revealed CD68^+^ cells, providing protein-level evidence for the presence of macrophage-like cells (Supplementary Material S4), consistent with ultrastructural morphology (Figures 2c,X). Together, These findings corroborate the cellular heterogeneity identified by scRNA-seq and support the multi-lineage nature of the model.

### 3.4 TGF-β1 induces fibrotic phenotype, epithelial barrier disruption, ferroptosis-like mitochondrial alterations, and fibrotic remodeling in the fibrosis model

**FIGURE 4:**
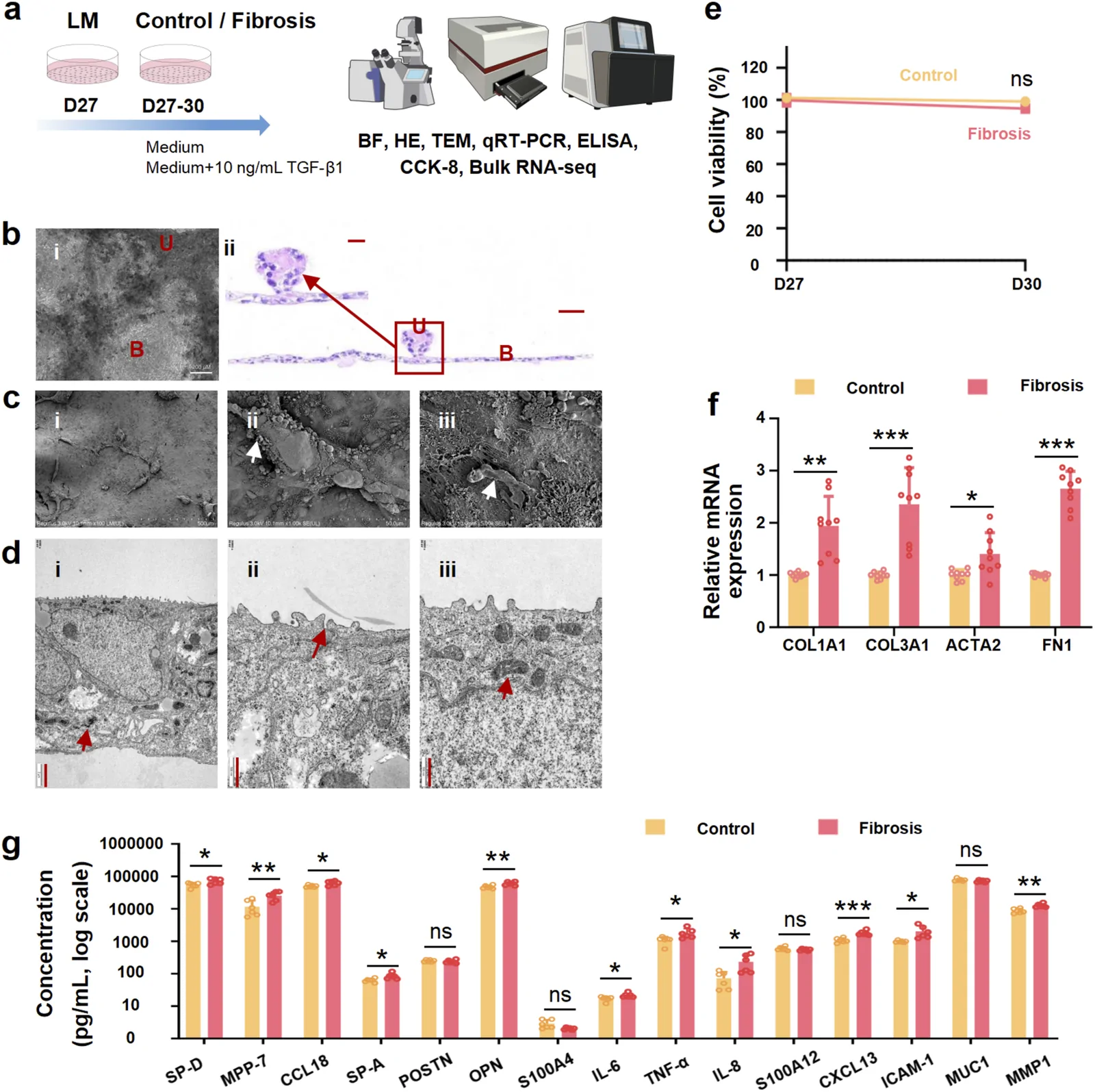
TGF-β1 induces fibrotic phenotype, epithelial barrier injury, and ferroptosis features in the lung model. **(a)** Schematic of the experimental workflow for TGF-β1 (10 ng/mL, 72 h) stimulation. **(b)** Bright-field images and H&E-stained longitudinal sections before and after TGF-β1 stimulation. B: basal layer; U: upper layer. Bright-field scale bar: 200 μm; H&E scale bars: left 20 μm, right 50 μm. **(c)** SEM images after TGF-β1 stimulation. (i) Overall surface morphology; (ii-iii) thickened and densified fibrous network with sheet-like dense deposits and increased spherical granular secretions. Arrows: spherical granular secretions (ii), sheet-like dense deposits (iii). Scale bars: 500 μm, 50 μm, and 10 μm, respectively. **(d)** TEM images after TGF-β1 stimulation. (i-ii) Disrupted tight junctions and widened intercellular spaces; (iii) ferroptosis-like mitochondrial changes: reduced volume, loss of cristae, and increased electron density. Arrows: disrupted tight junctions (ii), mitochondria with ferroptosis-like changes (i, iii). Scale bars: 2 μm, 1 μm, and 500 nm, respectively. **(e)** CCK-8 cell viability assay. Data are presented as mean ± SEM. Statistical significance was determined by Student’s unpaired t-test (Control vs. Fibrosis). ns, not statistically significant. **(f)** qRT-PCR analysis of COL1A1, COL3A1, FN1, and ACTA2 mRNA expression, normalized to controls. Data are presented as mean ± SEM. Statistical significance was determined by Welch’s t-test (Control vs. Fibrosis). *p < 0.05, **p < 0.01, ***p < 0.001. **(g)** ELISA measurement of IPF-associated biomarkers (SP-D, SP-A, MMP-7, MMP-1, CCL18, CXCL13, IL-6, IL-8, TNF-α, OPN, ICAM-1, POSTN, S100A4, S100A12, and MUC1) secretion levels (log scale). Statistical significance was determined by Student’s unpaired t-test (Control vs. Fibrosis), except for SP-D, POSTN, S100A4, IL-8, and ICAM-1, which were analyzed using Welch’s t-test. *p < 0.05, **p < 0.01 vs. control. ns, not statistically significant.

To simulate IPF pathology, day-27 multi-lineage lung models were treated with 10 ng/mL TGF-β1 for 72 h (Figure 4a). Histomorphologically, bright-field and H&E staining revealed that TGF-β1 treatment disrupted the original biphasic structure: the continuity of the upper loose reticular layer was interrupted, with dark punctate deposits appearing, the upper layer became condensed eosinophilic material, and cavity-like structures disappeared (Figure 4b). SEM observation showed that after treatment, the surface became flatter and denser, with fewer branched ridges and undulating textures. The fibrous network in intercellular spaces and on the surface became markedly thickened and densified, with filaments fusing into sheet-like dense deposits, accompanied by increased spherical granular secretions and decreased pore-like cavities (Figure 4c). At the subcellular level, TEM revealed disrupted tight junctions and markedly widened intercellular spaces after treatment; some epithelial mitochondria exhibited reduced volume, increased electron density, and decreased or absent cristae, consistent with ferroptosis-like ultrastructural changes (Figure 4d). CCK-8 assays showed no significant differences in cell viability among groups (Figure 4e), and LDH release assays revealed no significant disruption of membrane integrity (Supplementary Material S5).

At the molecular level, TGF-β1 treatment significantly upregulated *COL1A1*, *COL3A1*, *FN1*, and *ACTA2* mRNA in the fibrosis model compared with the controls (Figure 4f). *N-cadherin* (*CDH2*) mRNA was also significantly upregulated, whereas *Vimentin* (*VIM*) and S100A4 showed no significant changes (Supplementary Material S6). ELISA revealed significantly elevated secretion of multiple IPF-related proteins in fibrosis model group supernatants compared with the controls, including alveolar epithelial injury markers SP-D and SP-A; matrix remodeling enzymes MMP-7 and MMP-1; pro-inflammatory and profibrotic factors CCL18, CXCL13, IL-6, IL-8, TNF-α, and osteopontin (OPN); and the cell adhesion molecule ICAM-1. However, POSTN, S100A4, S100A12, and MUC1 levels showed no significant differences between groups (Figure 4g). These results indicate that TGF-β1 stimulation induces epithelial barrier disruption, ferroptosis-like mitochondrial alterations, ECM remodeling, and myofibroblast activation in the fibrosis model, capturing multiple key pathological features relevant to IPF.

### 3.5 Transcriptomic features of the fibrosis model are highly similar to human IPF

**FIGURE 5:**
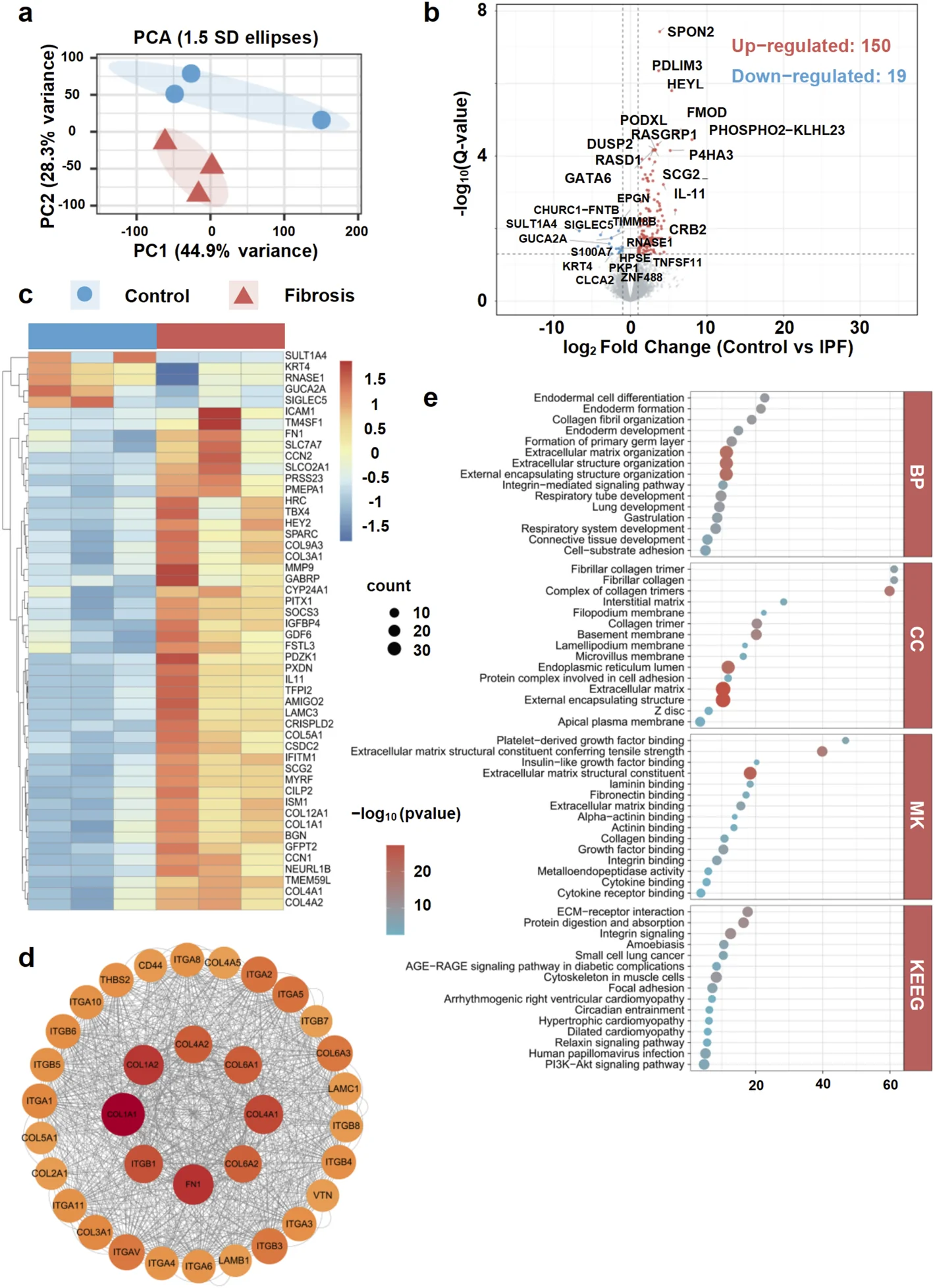
Transcriptomic analysis and GSEA validation. **(a)** Principal component analysis (PCA) plot. **(b)** Volcano plot of differentially expressed genes. **(c)** Heatmap of the top 50 differentially expressed genes ranked by variance. **(d)** Protein-protein interaction (PPI) network. **(e)** GO biological process and KEGG pathway enrichment analysis (top 15 upregulated terms).

To investigate the molecular pathology of the TGF-β1-induced fibrosis model, we performed RNA-seq on the samples. Principal component analysis (PCA) showed significant separation between control and fibrosis model groups along principal component 1 (PC1, explaining 44.9% of variance), with high intra-group clustering (Figure 5a), indicating robust grouping and biologically meaningful differences. Using |log_2_ FC| > 1 and FDR <0.05, 169 significantly differentially expressed genes (DEGs) were identified (150 upregulated, 19 downregulated; Supplementary Document S2). The volcano plot showed the distribution of these DEGs, with notable upregulation of *SPON2*, *PDLIM3*, *HEYL*, *FMOD*, *P4HA3*, *IL-11*, and *HPSE*, and downregulation of *SIGLEC5*, *SULT1A4*, *RNASE1*, and *KRT4* (Figure 5b). Notably, *CDH2* (N-cadherin) was significantly upregulated, while EMT-related markers, including *CDH1*, *CLDN1*, *DSP*, *OCLN*, *VIM*, S100A4, SNAI1, and SNAI2, did not reach statistical significance after FDR correction (Supplementary Document S2). Heatmap analysis of the top 50 variance-ranked DEGs further showed high expression of ECM-related genes (*FN1*, *COL1A1*, *COL4A1*/*COL4A2*, *MMP9)* and downregulation of SULT1A4 and KRT4 in the fibrosis model group (Figure 5c). Protein-protein interaction (PPI) network analysis (Figure 5d) revealed that *COL1A1*, *FN1*, *COL4A1*, *COL4A2*, *COL6A1*, *COL6A2*, and *ITGB1* formed core nodes, interacting with integrin family members (*ITGA1-5*, *ITGAV*, *ITGB3-4*, etc.) and laminins (*LAMC1*, *LAMB1*), suggesting widespread activation of ECM deposition and cell adhesion signaling. Gene Ontology (GO) and Kyoto Encyclopedia of Genes and Genomes (KEGG) enrichment analyses (Figure 5e) showed that upregulated genes were significantly enriched in biological processes such as extracellular matrix organization and cell adhesion, ECM-receptor interaction, focal adhesion, and PI3K-Akt signaling pathways, consistent with the PPI and heatmap results. Of note, no enrichment of EMT-related terms was observed in the GO/KEGG analyses for either upregulated or downregulated genes (Supplementary Document S3).

To evaluate transcriptomic similarity between the fibrosis model and human IPF, Gene Set Enrichment Analysis (GSEA) against Molecular Signatures Database (MSigDB) on the model group was performed. GSEA revealed that the top enriched pathways were ECM and collagen related, including extracellular matrix organization (NES = 2.749), degradation of the extracellular matrix (NES = 2.554), collagen formation (NES = 2.664), collagen degradation (NES = 2.556), and collagen biosynthesis and modifying enzymes (NES = 2.575) (Supplementary Material S7). Bidirectional GSEA validation was then performed using three independent GEO datasets (GSE17978, GSE47460, and GSE213001). First, IPF-associated upregulated genes from the three datasets showed significant positive enrichment in the model group (Supplementary Material S8), indicating transcriptomic consistency with human IPF. Second, upregulated genes from the model group showed significant positive enrichment in all three IPF cohorts: normalized enrichment score (NES) = 1.80 (p = 2.64e-04) for GSE17978, NES = 1.46 (p = 1.55e-02) for GSE47460, and a consistent trend for GSE213001 (NES = 1.97, p = 1.05e-04) (Supplementary Material S9), further supporting that upregulated genes from the model group are highly enriched in the transcriptomes of real IPF patients with significant clinical relevance.

Collectively, these results indicate that the fibrosis model is characterized by excessive ECM deposition, dysregulated cell adhesion, and activation of PI3K-Akt signaling. Genes including *FN1*, *COL1A1*, integrin family members, and *P4HA3* were significantly upregulated and occupied core positions in the PPI network, suggesting their important roles in fibrosis onset, and these changes were reproducible across independent IPF cohorts.

### 3.6 Pirfenidone reverses TGF-β1-induced fibrotic phenotypes

**FIGURE 6:**
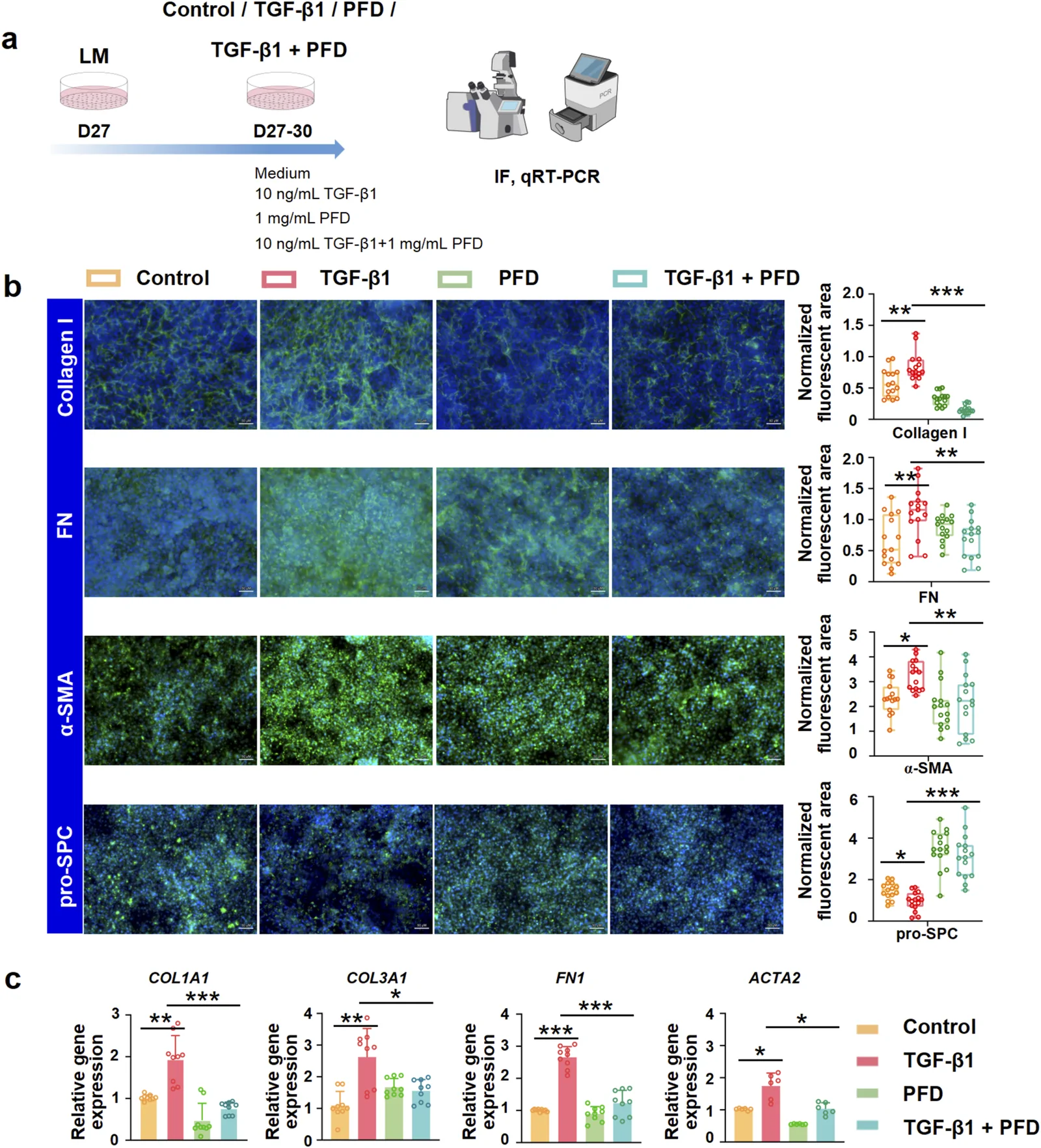
Pirfenidone reverses TGF-β1-induced fibrotic phenotypes. **(a)** Schematic of the experimental workflow for co-treatment with TGF-β1 (10 ng/mL) and pirfenidone (PFD, 1 mg/mL) for 72 h. Groups: control, PFD alone, TGF-β1 model, and TGF-β1+PFD intervention. **(b)** Representative immunofluorescence images showing the expression and distribution of collagen I, fibronectin, α-SMA, and Pro-SPC across groups. Scale bar: 50 μm. Quantification of immunofluorescence staining intensity for each marker. Data are presented as mean ± SEM. For multi-group comparisons, one-way ANOVA with Tukey’s HSD *post hoc* test was applied for fibronectin and α-SMA, while Welch’s ANOVA followed by Games-Howell *post hoc* test was used for collagen I and Pro-SPC (Control vs. TGF-β1 and TGF-β1 vs. TGF-β1+PFD). *p < 0.05, **p < 0.01, ***p < 0.001. **(c)** qRT-PCR analysis of COL1A1, COL3A1, FN1, and ACTA2 mRNA expression; Data are presented as mean ± SEM. For multi-group comparisons, while Welch’s ANOVA followed by Games-Howell *post hoc* test was used for COL1A1, COL3A1, FN1, and ACTA2 (Control vs. TGF-β1 and TGF-β1 vs. TGF-β1+PFD). *p < 0.05, **p < 0.01, ***p < 0.001.

To evaluate the utility of the model in drug evaluation, the models were co-treated with TGF-β1 (10 ng/mL) and the clinical drug pirfenidone (PFD, 1 mg/mL) for 72 h, with control, PFD-alone, TGF-β1 treatment, and TGF-β1+PFD co-treatment groups established (Figure 6a). Immunofluorescence analysis showed that TGF-β1 stimulation significantly enhanced collagen I and fibronectin signals, whereas PFD intervention substantially reduced their fluorescence intensity; the PFD-alone group showed no notable changes compared with the control group. qRT-PCR results were consistent with this, showing that PFD significantly inhibited TGF-β1-induced upregulation of *COL1A1*, *COL3A1*, and *FN1* mRNA (Figures 6b,c).

Regarding myofibroblast activation, qRT-PCR results showed that *ACTA2* mRNA levels in the model group were significantly upregulated compared with the control group, while the PFD intervention group showed a significant reduction; immunofluorescence further confirmed that α-SMA fluorescence signals were significantly enhanced in the model group compared with the control group and markedly attenuated in the PFD intervention group (Figures 6b,c). Additionally, TGF-β1 led to reduced Pro-SPC signals (an AT2 cell marker), which were partially restored by PFD intervention, suggesting a protective effect of PFD on AT2 cells.

These results indicate that the clinical drug PFD effectively reverses TGF-β1-induced excessive ECM deposition and myofibroblast activation in this model, and partially restores AT2 cell marker expression, with no apparent effects on control models when administered alone, indicating that the fibrosis model can serve as an *in vitro* platform for antifibrotic drug screening and efficacy evaluation.

## 4 Discussion

In this study, a humanized multi-lineage lung model was established through directed differentiation of hiPSC, which endogenously generates proximal duct-like epithelium, distal alveolar epithelial, fibroblasts, endothelial cells, and CD68^+^ macrophage-like cells within a single system. Multi-dimensional characterization confirmed this multi-lineage composition and suggested epithelial-mesenchymal co-development. TGF-β1 treatment induced fibrosis-like phenotypes including epithelial barrier disruption, ferroptosis-like mitochondrial alterations, and ECM remodeling, with transcriptomic features highly consistent with human IPF and drug responsiveness confirmed for PFD. This platform provides a humanized *in vitro* tool for IPF research and drug screening.

Compared with existing lung models, this system generates multiple lineages without exogenous cell supplementation. Kotton and colleagues generated proximal airway organoids or alveolar spheres, but each was limited to a single lineage (McCauley et al., 2017; Jacob et al., 2017). Hawkins et al. obtained proximal airway epithelium, but lacked distal and mesenchymal components (Berical et al., 2022). Spence laboratory generated lung organoids containing proximal epithelium and mesenchyme, but these models lacked endothelial and immune cells and exhibited immature differentiation (Dye et al., 2015). Snoeck laboratory generated lung bud organoids that formed branching airways and early alveolar structures, but required xenotransplantation and lacked endothelial and immune cells (Chen et al., 2017). None of these systems achieved endogenous co-development of proximal epithelium, distal epithelium, mesenchyme, endothelial cells, and immune cells within a single system. The synchronous emergence of multiple lineages suggests that early lung progenitor cells may retain mesenchymal differentiation potential, or that an as-yet-unidentified common progenitor may exist. One possible mechanism involves dynamic modulation of BMP signaling (Weaver et al., 1999; Suzuki et al., 2025): transient inhibition of BMP to relieve its constraint on endodermal regionalization, followed by restoration of BMP activity to synergize with FGF and retinoic acid signaling, thereby promoting proximal-distal axis establishment and mesenchymal progenitor maintenance. This dynamic modulation temporally mimics the spatiotemporal expression pattern of BMP during *in vivo* lung development (Jacob et al., 2017; Miao et al., 2025), creating a broader permissive window for multi-lineage establishment. However, this hypothesis requires direct experimental validation, and the precise origin of mesenchymal cells in this model remains to be determined. Nevertheless, the ability of this model to synchronously generate multiple lineages remains of significant research value. Thus, this model serves as a complementary platform for studying epithelial-mesenchymal co-development mechanisms.

TGF-β1 stimulation induced marked structural alterations, including disruption of the epithelial-stromal bilayer and thickening of ECM fiber networks. TEM revealed epithelial barrier disruption and specific mitochondrial changes, including loss of tight junction, widened intercellular spaces, and mitochondria with reduced volume, decreased or absent cristae, and increased electron density, displaying ferroptosis-like ultrastructural features (Chen et al., 2021; Stockwell, 2022). These changes occurred without significant loss of cell viability or membrane integrity, suggesting early ferroptotic stress rather than terminal ferroptosis, consistent with the established ferroptosis cascade, in which mitochondrial shrinkage and cristae loss precede plasma membrane rupture (Stockwell, 2022). Ferroptosis is an iron-dependent, lipid peroxidation-driven form of regulated cell death. Previous studies have linked ferroptosis to fibrosis progression (Pei et al., 2022; Song et al., 2025). In this model, mitochondrial ferroptosis-like changes co-occurred with upregulation of *ACTA2*, *COL1A1*, *FN1*, and *CDH2*, supporting a link between ferroptosis and fibrotic activation. These findings are consistent with (Wu et al., 2024), who also reported *CDH2* upregulation and identified FN1 and COL1A1 as PPI hub genes. At the transcriptomic level, FMT markers (ACTA2, COL1A1, FN1) were significantly upregulated, whereas EMT-related markers (*VIM*, S100A4, SNAI1, SNAI2) showed no significant changes, indicating that classical EMT is not activated and supporting FMT as the dominant fibrotic mechanism in this model. RT-PCR analysis of selected markers (*CDH2*, *VIM*, S100A4) was consistent with these transcriptomic findings. Future experiments with ferroptosis inhibitors (e.g., ferrostatin-1, liproxstatin-1) could further validate the causal relationship.

Despite the low proportion (2.31%) of fibroblasts in this model, TGF-β1 stimulation still induced significant ECM deposition and myofibroblast activation. Fibroblast characteristics in fibrosis lie in functional state changes rather than numerical increases (Tsukui et al., 2013). Even small numbers of activated fibroblasts can amplify their activity through paracrine signaling and recruit neighboring cells (Epstein Shochet et al., 2020). Importantly, this paracrine crosstalk is not unidirectional—bidirectional signaling between epithelial and mesenchymal cells has been recognized as a core feature of the profibrotic microenvironment (Yao et al., 2021). These observations suggest that the small number of fibroblasts in this model is sufficient to drive fibrotic responses, possibly through paracrine coordination with other cell types. In single-cell sequencing, cluster 5 (partial EMT epithelium, partial AT2-like) co-expressed epithelial (EPCAM) and mesenchymal (COL1A1, FN1) markers. GO enrichment analysis showed mitochondrial energy metabolism and ribosome biogenesis, without EMT or inflammation enrichment, supporting its identity as a transitional cell state rather than a pathological EMT product. Partial EMT can promote fibroblast activation and ECM deposition through paracrine mechanisms (Hill et al., 2019; Yao et al., 2021; Bastos et al., 2025), suggesting that cluster 5 may coordinate with fibroblasts in fibrotic responses. However, its physiological versus pathological role requires further investigation.

The FMT-driven ECM remodeling was further validated at the secretory protein level. ELISA showed significant elevation of IPF-associated biomarkers including MMP-7 and SP-D, CCL18, CXCL13, IL-6, IL-8, TNF-α, and osteopontin (Rosas et al., 2008; Prasse et al., 2006; Vuga et al., 2014; Suri et al., 2024; Pardo et al., 2005; Zhu et al., 2024), consistent with the IPF secretomic signature. No significant changes were observed for POSTN, S100A4, S100A12, or MUC1, whose regulation involves IL-13/IL-4 or TLR/RAGE pathways in addition to TGF-β signaling (Rose et al., 2000; Ashley et al., 2017; Meng et al., 2025; Sun et al., 2025; Tanaka et al., 2019; Borchert et al., 2025; Raveendran et al., 2025). This selective non-responsiveness suggests that the model may be useful for dissecting the differential effects of distinct profibrotic factors and their pathway interactions, rather than merely mimicking all known clinical biomarker changes.

Transcriptomic analysis further revealed enrichment in ECM organization, cell adhesion, and PI3K-Akt signaling pathways, mutually reinforcing the upregulation of MMP-7 and FN1 in the secretory profile, and consistent with ECM over-deposition and persistent fibroblast activation,in IPF (Bhatt et al., 2025). Bidirectional GSEA using three independent IPF cohorts further confirmed the transcriptomic reproducibility of the model in human IPF (Hu and Xu, 2024). Consistent with these findings, PFD responsiveness confirmed the model’s utility for detecting pharmacologically reversible fibrotic changes, and PFD’s pleiotropic mechanisms (Ruwanpura et al., 2020; Knüppel et al., 2017) suggest its potential for systematic drug evaluation.

Several limitations should be acknowledged. First, the abundance and maturity of individual lineages require improvement. AT2-like cells accounted for only 0.64%, endothelial cells for 1.15%, and fibroblasts for 2.31%. Macrophage-like cells were not resolved as an independent cluster, and no other immune cell clusters were identified. The proximal duct-like epithelium similarly lacked mature basal, club, or ciliated cells. These limitations likely reflect current culture conditions: the 27-day differentiation window may be sufficient for early lineage specification but inadequate for terminal maturation of certain populations—particularly AT2 cells, which typically require prolonged culture to acquire mature functional characteristics. Moreover, the submerged culture system lacks air-liquid interface (ALI) and mechanical stimuli, which are known to influence epithelial polarization, ciliogenesis, and terminal differentiation of airway epithelial cells (Kim et al., 2024; Walls et al., 2024). The absence of myeloid differentiation signals (e.g., GM-CSF, M-CSF) and inflammatory stimuli also restricts immune cell differentiation and maturation, as GM-CSF supports macrophage tissue adaptation (Kang et al., 2025), and IFN-γ exerts stage-dependent regulatory effects on lung organoids cell populations (Dost et al., 2026). Future optimization could address these limitations through extended culture, introduction of ALI or microfluidic systems, cytokine supplementation (Seo et al., 2022; McVicar et al., 2024; Jo et al., 2024), or integration of spatial transcriptomics to better capture rare populations. Second, fibrosis was induced by TGF-β1 alone; whereas the IPF microenvironment involves multiple factors including PDGF, Wnt, TNF-α, and matrix stiffness (Gao et al., 2025; Nieves and García, 2025). Thus, this model represents a TGF-β1-induced fibrosis-like system that captures key fibrotic processes relevant to IPF, rather than the full disease. Future incorporation of multi-factor combinations could more comprehensively simulate the complex IPF microenvironment. Third, scRNA-seq capture bias for rare populations persists (Ye et al., 2025; Nesari et al., 2026), which may account for the absence of macrophage-like cells as an independent cluster. Future integration with single-nucleus sequencing or spatial transcriptomics could complement this limitation. Additionally, hiPSC lines exhibit inherent variability in differentiation propensity and response thresholds (Lancaster and Huch, 2019; Gaffey et al., 2025); whether the observed multi-lineage characteristics are generalizable to other cell lines requires further validation.

## 5 Conclusion

In this study, a humanized multi-lineage lung model was established based on directed differentiation of hiPSCs. Through temporal modulation of signaling pathways, and without genetic editing or exogenous cell supplementation, this model achieves generation of proximal and distal epithelial structures, endogenously integrating fibroblasts, endothelial cells, and macrophage-like cells, thereby providing an experimental platform for studying epithelial-mesenchymal co-development in the lung. TGF-β1 stimulation induces epithelial barrier disruption, ferroptosis-like mitochondrial ultrastructural alterations, ECM remodeling, and aberrant secretion of multiple IPF-associated biomarkers. Bidirectional GSEA validation confirmed that the transcriptomic signature of the model shows high similarity to human IPF, and PFD effectively reverses these fibrotic phenotypes. This platform provides a humanized and standardizable tool for IPF pathogenesis research and antifibrotic drug screening. Its multi-lineage self-assembly properties and clinical pathological relevance lay a foundation for investigating multicellular interaction networks in IPF and the development of novel therapeutic strategies.

## Data Availability

Publicly available datasets were analyzed in this study. This data can be found here: The RNA-seq and scRNA-seq data generated in this study are available from the corresponding author upon reasonable request. The publicly available datasets GSE17978, GSE47460, and GSE213001 were analyzed in this study and are available in the GEO database (https://www.ncbi.nlm.nih.gov/geo/).
