# Supplementary material for: An hiPSC-derived multi-lineage lung model exhibiting proximal-distal epithelial features for modeling pulmonary fibrosis
Source: Front Cell Dev Biol. 2026 Aug 25;14:1914941. doi: 10.3389/fcell.2026.1914941 (PMC13550129; doi:10.3389/fcell.2026.1914941)
Supplement: Supplementary file 1 [file DataSheet1.zip › Supplementary File/Supplementary Code Materials/Readme_Supplementary Code Materials.docx]

**Supplementary Code Materials**

This supplementary code materials document contains R scripts and corresponding data files used to generate Figures 3 and 5, as well as Supplementary Information 2–3 and 7–9.

**1. Single-Cell RNA-seq Data Analysis**

(Corresponding to Figures 3a–c; Supplementary Information 2 and 3)

Folder: Figure 3a 3b 3c Supplementary Information 2 3

The large RDS data file (seurat_integrated_annotated_v2.rds) has been deposited

in Figshare and is available at: https://doi.org/10.6084/m9.figshare.33087275

**File Inventory**

**1.1 Figure 3a 3b Supplementary Information 2 3.R**

- R script for generating Figure 3a (UMAP plot) and Figure 3b (cell-proportion bar chart).
- Also generates Supplementary Information 2 (Feature plots) and Supplementary Information 3 (GO enrichment bubble plots).
- Required R packages: Seurat, ggplot2, clusterProfiler, etc.
- Input data: seurat_integrated_annotated_v2.rds

**1.2 Figure 3c.R**

- R script for generating Figure 3c (pseudotime trajectory analysis).
- Required R packages: Monocle 3, Slingshot, etc.
- Input data: seurat_integrated_annotated_v2.rds

**1.3 seurat_integrated_annotated_v2.rds**

- Processed Seurat object from single-cell RNA-seq data.
- Contains annotation information and UMAP coordinates for 21,132 cells.
- Load in R using the readRDS() function.

**2. Transcriptomic Data Analysis**

(Corresponding to Figures 5a–c and Figure 5e data)

Folder: Figure 5a 5b 5c and data 5e

**File Inventory**

**2.1 Figure 5a.R**

- R script for generating Figure 5a (PCA plot).
- Required R packages: DESeq2, ggplot2
- Input data: C vs T TPM.xlsx

**2.2 Figure 5b.R**

- R script for generating Figure 5b (volcano plot).
- Required R packages: DESeq2, ggplot2
- Input data: C vs T FDR.xlsx

**2.3 Figure 5c.R**

- R script for generating Figure 5c (heatmap showing top 50 differentially expressed genes).
- Required R packages: DESeq2, pheatmap
- Input data: C vs T TPM.xlsx

**2.4 Figure 5e.R**

- R script for generating Figure 5e (GO and KEGG enrichment lists).
- Required R packages: clusterProfiler, org.Hs.eg.db, DOSE
- Input data: 150 up.csv, 19 down.csv

**2.5 C vs T TPM.xlsx**

- TPM expression matrix (Control vs TGF-β1-treated group).
- Expression levels for all genes across all samples.

**2.6 C vs T FDR.xlsx**

- Differential expression analysis results.
- Contains: GeneID, log_2_FC, Q_value, P_value

**2.7 150 up.csv**

- Differential expression results of upregulated genes.
- Contains: gene_symbol	, diffexp_log2fc, diffexp_deseq2_qvalue, diffexp_deseq2_pvalue

**2.8 19 down.csv**

- Differential expression results of downregulated genes.
- Contains: gene_symbol	, diffexp_log2fc, diffexp_deseq2_qvalue, diffexp_deseq2_pvalue

**3. GSEA Validation**

(Corresponding to Supplementary Information 7, 8, and 9)

Folder: Supplementary Information 7 8 9

**File Inventory**

**3.1 7 GSEA.R**

- R script for performing Gene Set Enrichment Analysis (GSEA) and generating a three-panel composite figure (enrichment score curves, gene ranking metrics, and gene-set membership markers).
- Required R packages: readxl, dplyr, fgsea, ggplot2, patchwork, msigdbr
- Input data: GSEA.xlsx

**3.2 8.R**

- R script for performing GSEA enrichment analysis by comparing three publicly available IPF up-regulated gene sets (GSE17978, GSE47460, GSE213001) against the model transcriptomic data, to validate the similarity between the model and clinical data.
- Required R packages: ggplot2, fgsea, dplyr, tidyr, readxl
- Input data: GSEA.xlsx, GSE17978-up.csv, GSE47460-up.csv, GSE213001-up.csv

**3.3 9.R**

- R script for performing cross‑validation GSEA using the model up‑regulated gene set against three independent clinical IPF datasets (GSE213001, GSE47460, GSE17978).
- Required R packages: ggplot2, fgsea, dplyr, tidyr
- Input data: 150 up.csv, GSE213001 xin.csv, GSE47460 xin.csv, GSE17978 xin.csv

**3.4 GSEA.xlsx**

- Transcriptomic differential expression results (Excel format).
- Contains: gene_symbol, diffexp_log2fc, diffexp_deseq2_pvalue, diffexp_deseq2_qvalue

**3.5 GSE17978-up.csv**

- Up-regulated gene list extracted from the GSE17978 dataset.
- Contains: gene_symbol, log2FC, pvalue, padj

**3.6 GSE47460-up.csv**

- Up-regulated gene list extracted from the GSE47460 dataset.
- Contains: gene_symbol, log2FC, pvalue, padj

**3.7 GSE213001-up.csv**

- Up-regulated gene list extracted from the GSE213001 dataset.
- Contains: gene_symbol, log2FC, pvalue, padj

**3.8 GSE17978 xin.csv**

- Differential expression results from the GSE17978 dataset.
- Contains: gene_symbol, log2fc, pvalue, padj

**3.9 GSE47460 xin.csv**

- Differential expression results from the GSE47460 dataset.
- Contains: gene_symbol, log2fc, pvalue, padj

**3.10 GSE213001 xin.csv**

- Differential expression results from the GSE213001 dataset.
- Contains: gene_symbol, log2fc, pvalue, padj

**Usage Instructions**

Before running any R script, ensure that all files are placed in the same working directory.

Place the data files from the corresponding folder into the directory where the script is located.

The scripts will automatically read the data files and generate the corresponding figures/data.

For questions regarding the code or data, please contact the corresponding author.
