# Supplementary material for: An hiPSC-derived multi-lineage lung model exhibiting proximal-distal epithelial features for modeling pulmonary fibrosis
Source: Front Cell Dev Biol. 2026 Aug 25;14:1914941. doi: 10.3389/fcell.2026.1914941 (PMC13550129; doi:10.3389/fcell.2026.1914941)

Title Page

Type of contribution: Research paper

Title: An hiPSC-derived multi-lineage lung model recapitulates proximal-distal epithelial patterning to model pulmonary fibrosis

Running title: hiPSC-derived lung model for IPF

Author List and Affiliations: Chenfeng Hua[1,2†*], Chaojie Qu[1†], Liu Yang[1], Wenfei Zhao[3], Junwei Zhao[2], Yongliang Jia[4], Chengjie Ma[1], Pingping Shang[1], Xiang Li[2], Fuwei Xie[1], Cong Nie[1]

[†] These authors contributed equally to this work and share first authorship.

[1] Key Laboratory of Tobacco Chemistry, Zhengzhou Tobacco Research Institute of CNTC, Zhengzhou, China

[2] Beijing Life Science Academy, Beijing, China

[3] Department of Respiratory and Critical Care Medicine, Fifth Affiliated Hospital of Zhengzhou University, Zhengzhou, China

[4] Henan Institute of Medical and Pharmaceutical Sciences, Zhengzhou University, Zhengzhou, China

Corresponding author:

Hua Chenfeng, e-mail: niya_lucky@126.com

Supplementary Information 1

Supplementary Table S1: Reagents for human induced pluripotent stem cells (hiPSCs) culture.

Table S1. Reagents for hiPSCs culture

Compound	Company	Cat#	Stock [c]	Final [c]
Y-27632	Sigma	SCM075	10mM	10μM
mTeSR™ medium	STEMCELL	85851	-	-
Matrigel	Corning	354277	-	-
Accutase	Gibco	A1110501	-	-
Trypan Blue	Gibco	15250061	-	-
DPBS	Gibco	14190-144	-	-

Supplementary Table S2: Composition of the basal differentiation medium.

Table S2. Composition of the basal differentiation medium

Compound	Company	Cat#	Stock [c]	Final [c]
DMEM/F-12，GlutaMAX	Gibco	10565018	-	-
B-27 (50X)	Gibco	17504044	50 X	1 X
N-2 (100X)	Gibco	17502048	100 X	1 X
penicillin-streptomycin	Gibco	15140122	10,000 U/mL	50 U/mL
fetal bovine serum	Gibco	10100147	-	2%
L-Ascorbic acid	R&D systems	4055/50	100 mg/mL	50 μg/mL
Monothioglycerol	Sigma-Aldrich	M6145	0.15 mol/mL	400 μM

Supplementary Table S3: Factors for definitive endoderm (DE) differentiation.

Table S3. Factors for DE differentiation

Compound	Company	Cat#	Stock[c]	Final [c]	Time
rhBMP4	R&D systems	314-BPE-010	100 μg/mL	3 ng/mL	day 1
Y-27632	Sigma	SCM075	10 mM	10 μM	day 1,2
Activin A	R&D systems	338-AC-050	100 μg/mL	100 ng/mL	day 2-4
CHIR99021	Selleck	S2924	5 mM	1 μM	day 2-4

Supplementary Table S4: Factors for anterior foregut endoderm (AFE) differentiation.

Table S4. Factors for AFE differentiation

Compound	Company	Cat#	Stock [c]	Final [c]	Time
SB431542	Tocris bioscience	1614	50 mM	10 μM	day 5-7
Noggin	R&D systems	6057-NG-100	250 μg/mL	100 ng/mL	

Supplementary Table S5: Factors for lung progenitor (LPA) differentiation.

Table S5. Factors for LPA differentiation

Compound	Company	Cat#	Stock [c]	Final [c]	Time
CHIR99021	Selleck	S2924	5 mM	3 μM	day 8-17

rhBMP-4 (E. coli)	R&D systems	314-BPE-010	100 μg/mL	10 ng/mL	
all trans retinoic acid (ATRA)	Sigma-Aldrich	R2625	1 mM	1 μM	
rhKGF/FGF-7	R&D systems	251-KG-010	100 μg/mL	10 ng/mL	
rhFGF-10	R&D systems	345-FG-025	100 μg/mL	10 ng/mL	
rhEGF	R&D systems	236-EG-200	100 μg/mL	20 ng/mL	
rhFGF-2	abcam	ab179489	100 μg/mL	2 ng/mL	

Supplementary Table S6: Factors for human induced pluripotent stem cell-derived alveolar organoids (hiPSC-AOs) differentiation.

Table S6. Factors for hiPSC-AOs differentiation

Compound	Company	Cat#	Stock [c]	Final [c]	Time
CHIR99021	Selleck	S2924	5 mM	3 μM	day 18-27
rhKGF/FGF-7	R&D systems	251-KG-010	100 μg/mL	10 ng/mL	
rhFGF-10	R&D systems	345-FG-025	100 μg/mL	10 ng/mL	
Dexamethasone	Sigma-Aldrich	D4902-100MG	0.1 mM	50 nM	
8-Br-cAmp	Sigma-Aldrich	B5386	0.1 M	0.1 mM	
IBMX	Sigma-Aldrich	I5879	0.5 M	0.1 mM	
ITS	BioCoat	354350	100 %	0.05 %	

Supplementary Table S8: Primer sequences used for qRT-PCR.

Table S8. Primer sequences used for qRT-PCR

Gene	Forward (5'to 3')	Reverse (3'to 5')
β-actin	CATGTACGTTGCTATCCAGGC	CTCCTTAATGTCACGCACGAT
SOX17	ATACGCCAGTGACGACCAGA	TTCCACGACTTGCCCAGCAT
CXCR4	GCTGTTGGCTGAAAAGGTGG	ATCTGCCTCACTGACGTTGG
NKX2.1	GTACAGGCAGGGCCAGG	ATGAAGCGGGAGATGGCG
SOX2	ATGGACAGTTACGCGCACAT	CGAGCTGGTCATGGAGTTGT
SOX9	AGGAAGTCGGTGAAGAACGG	CTGGGATTGCCCCGAGTG
SFTPB	CTGGACAGGGAAAAGTGCAAGC	TCTCATCAAAGGTCGGGGCT
SFTPC	ACACACGGAGATGGTTCTGG	GGCCTGGAAGTTGTGGACTT
CPM	GCTGTTGCTGCCTTTGGTAG	CCGCCCAACAGTCTCATCTC
α-SMA	AGCCAAGCACTGTCAGGAAT	CACCATCACCCCCTGATGTC
FN1	TCCGGGACTCAATCCAAATGC	TCAAGCCTTCGTTGACAGAGT
COL1A1	GGAATGAAGGGACACAGAGGTT	GGCAGCACCAGTAGCACC
COL3A1	TAAAGGCGAAATGGGTCCCG	GGCACCATTCTTACCAGGCT

N-cadherin	AAGGACAGCCTCTTCTCAATGT	TCAACTTCTGCTGACTCCTTCA
Vimentin	AAGGCGAGGAGAGCAGGATT	TGGGTATCAACCAGAGGGAGT
S100A4	CGCTTCTTCTTTCTTGGTTTGATCC	ACTTGTCACCCTCTTTGCCC

Supplementary Table S7: Antibodies used for immunofluorescence staining.

Table S7. Antibodies used for immunofluorescence staining

Compound	Company	Cat#	Dilution
Anti-SOX2	Abcam	ab92494	1:100
Anti-SOX17	Abcam	ab224637	1:2000
Anti-CXCR4	Abcam	ab181020	1:500
Anti-FOXA2	Abcam	ab108396	1:500
Anti-SOX9	Abcam	Ab185966	1:200
Anti-NKX2.1	Abcam	ab227652	1:25
Anti-CD68	Cell Signaling Technology	76437	1:400
Anti-Pro-SPC	Abcam	ab90716	1:1000
Anti-Collagen I	Abcam	ab138492	1:1000
Anti-alpha smooth muscle Actin a (α-SMA)	Abcam	ab7817	1:50
Anti-Fibronectin (FN)	Abcam	ab268020	1/250
DAPI (Hydrochloride)	STEMCELL	75004	10 μg/mL
Goat Anti-Rabbit IgG H&L488	Abcam	ab150077	1:500
Goat Anti-Mouse IgG H&L488	Abcam	ab150113	1:500
Goat Anti-Rat IgGH&L488	Abcam	ab150157	1:500
Goat Anti-Rabbit IgG H&L647	Abcam	ab150079	1:500

Supplementary Table S9: ELISA kits used for quantification.

Table S9. ELISA kits used for quantification

Compound	Company	Cat#
Human SP-A	Dogesce	DG10666H
Human MMP-1	Dogesce	DG11386H
Human MMP-7	Dogesce	DG11381H
Human SP-D	Dogesce	DG10659H
Human CCL18	Dogesce	DG95671Q
Human OPN	Dogesce	DG10095H
Human CXCL13	HUABODEYI	HBDY96T-8624
Human ICAM-1	JiangLaiBio	JL13457
Human IL-8	ThermoFischer	D8000C
Human IL-6	ThermoFischer	D6050B
Human TNF-α	ThermoFisher	KHC3014

POSTN	HUABODEYI	HBDY96T-6040
MUC1	Dogesce	DG94344Q
Human TGF-β	HUABODEYI	HBDY96T-6482
Human S100A12	HUABODEYI	HBDY96T-6714
Human S100A4	HUABODEYI	HBDY-51127H1
Human TGF-β1	HUABODEYI	HBDY96T-6483

Supplementary Information 2

Feature plots of marker genes for each subcluster.

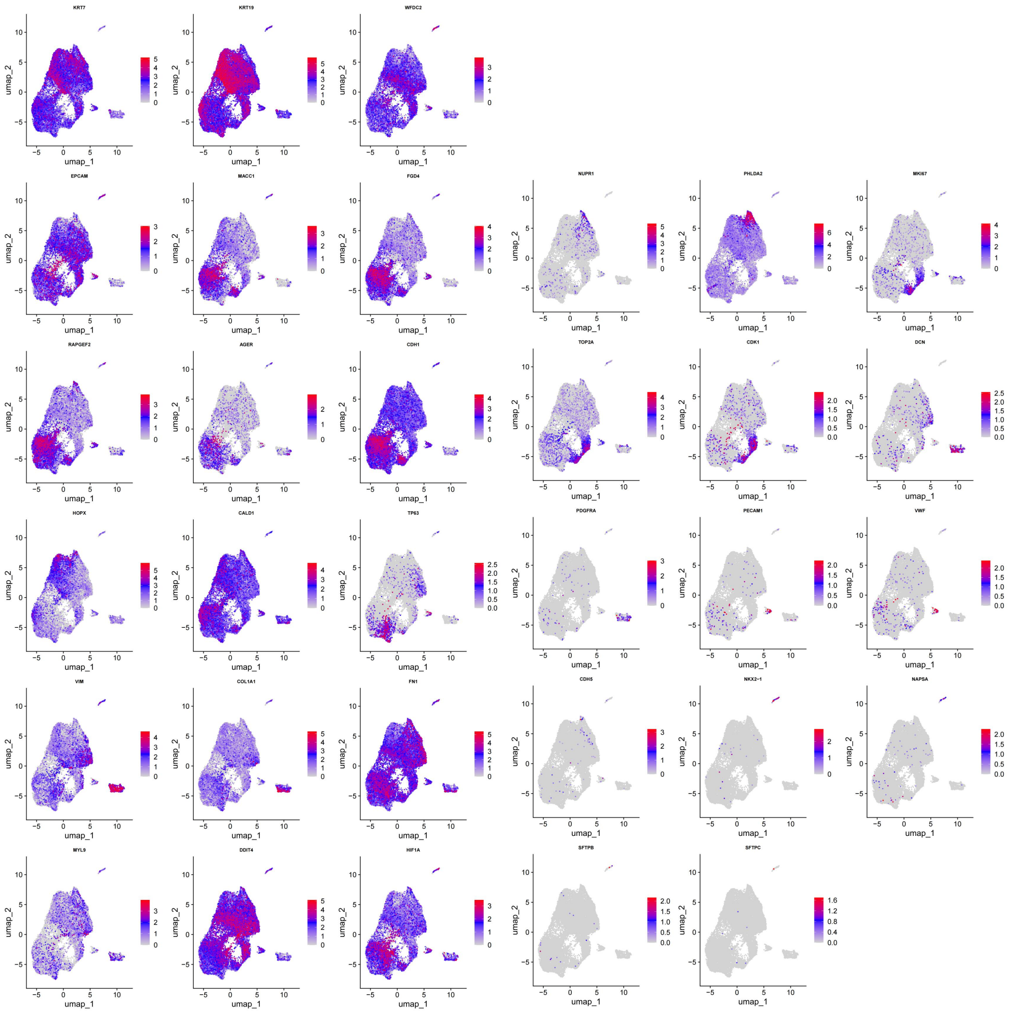

Supplementary Information 3

Bubble plots of GO BP enrichment analysis for each subcluster.

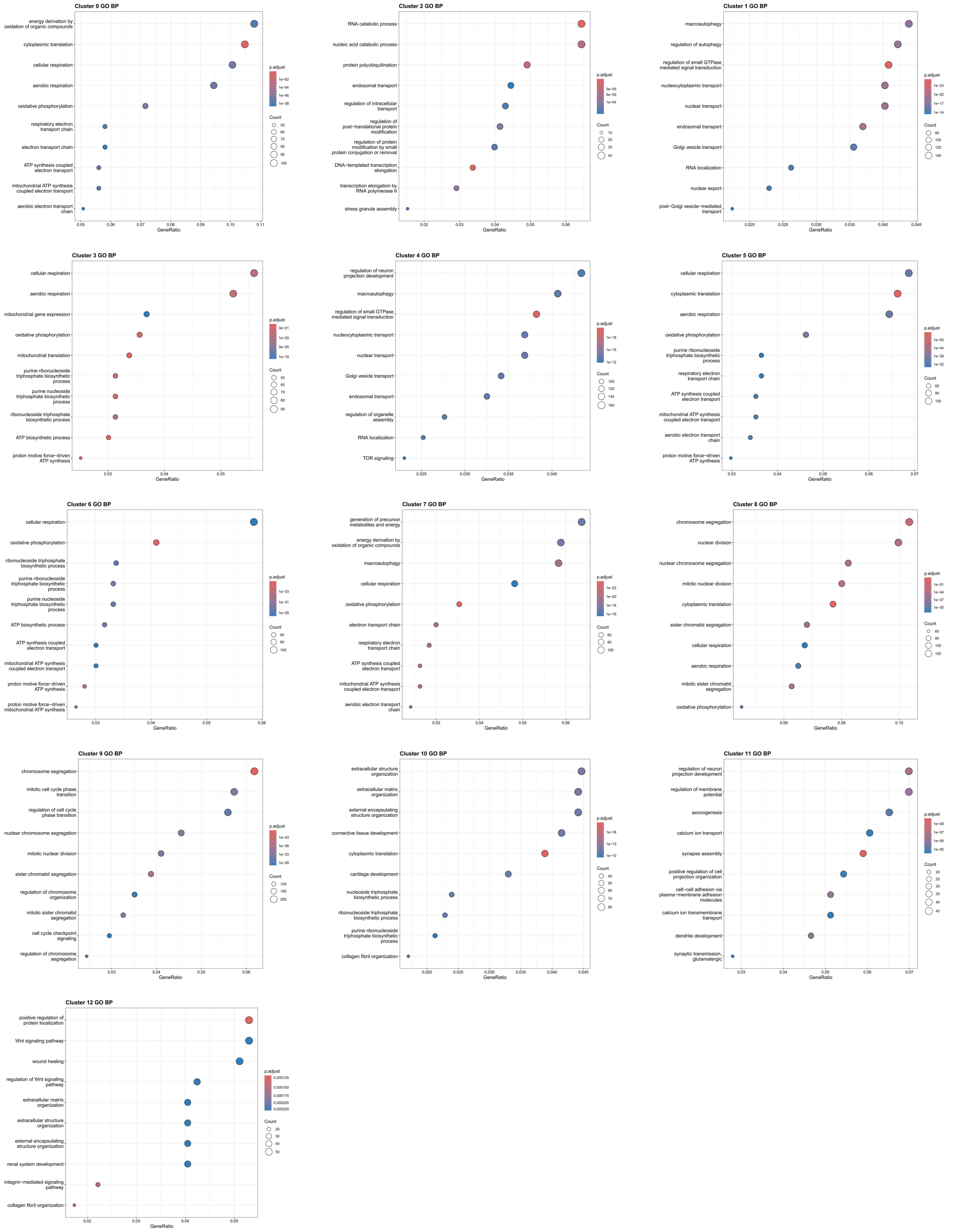

Supplementary Information 4

Immunofluorescence detection of CD68$^+$ macrophage-like cells and Pro-SPC$^+$ AT2-like cells.

(a) CD68$^+$ cells (green) in dissociated cells from day-27 models. Cells were prepared by cytospin, dropped onto poly-L-lysine-coated slides, and allowed to settle by gravity, then fixed and stained as described in Methods 2.5. Nuclei were counterstained with DAPI (blue). Scale bars: 50 μm.

(b) Pro-SPC$^+$ cells (red) on paraffin-embedded cross-sections (4 μm) from day-27 models. Sections were deparaffinized, rehydrated, and subjected to antigen retrieval, then stained as described in Methods 2.5. Pro-SPC-positive cells exhibit characteristic granular or perinuclear staining patterns (arrows), consistent with lamellar body distribution in AT2 cells. Nuclei were counterstained with DAPI (blue). Scale bars: 50 μm.

a

b

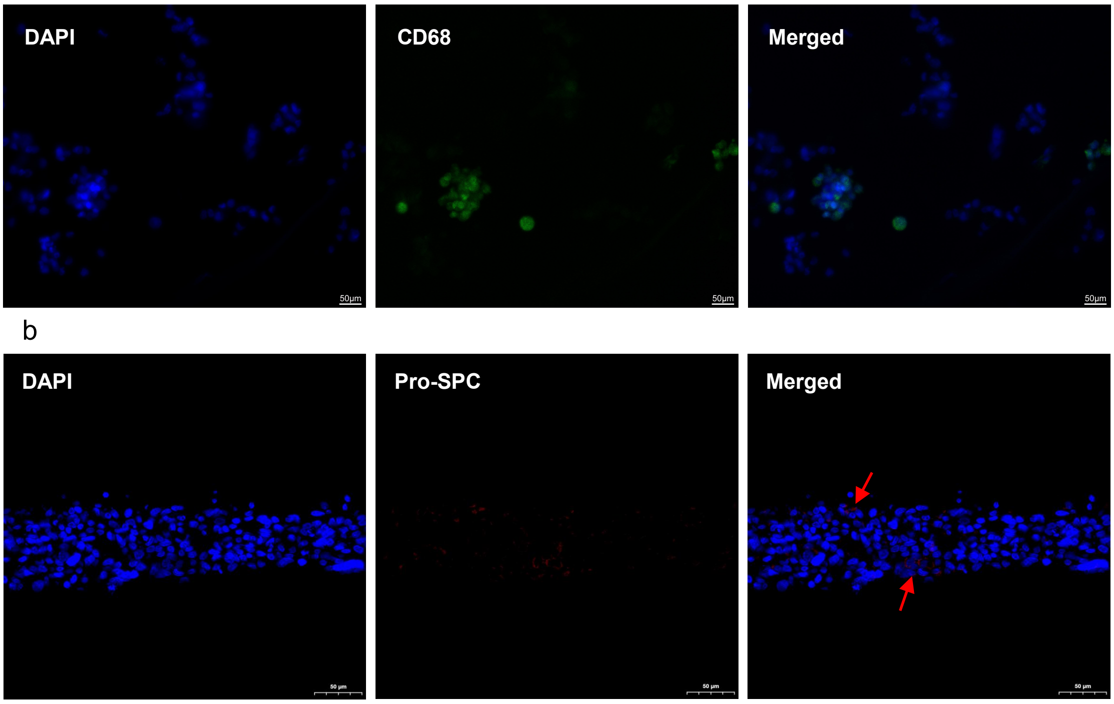

Supplementary Information 5

LDH release assay in the control and fibrosis model. Data are presented as mean ± SEM. Statistical significance was determined by Student's unpaired t-test (Control vs. Fibrosis). ns, not significant.

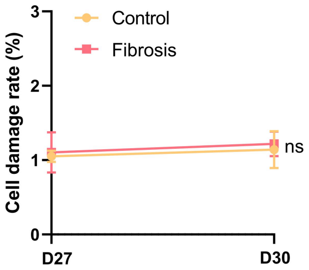

Supplementary Information 6

mRNA expression of *CDH2*, *VIM*, and *S100A4* in the control and fibrotic model. Data are presented as mean ± SEM. Statistical significance was determined by Student's unpaired t-test. ** $p < 0.01$; ns, not statistically significant.

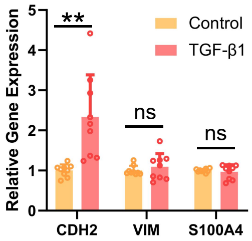

Supplementary Information 7

MSigDB-based GSEA showing enrichment curves for ECM-receptor interaction, focal adhesion, PI3K-Akt, TGF-β, and EMT pathways in the model group.

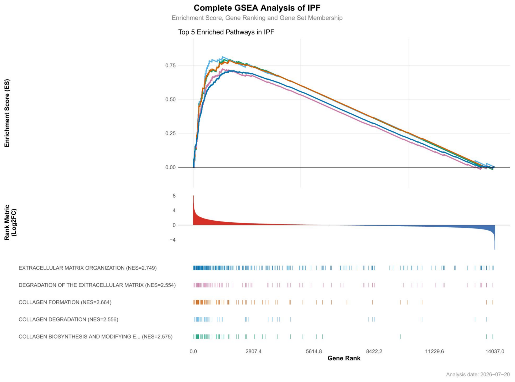

Supplementary Information 8

Bidirectional GSEA validation—enrichment of known IPF upregulated gene sets in the model group.

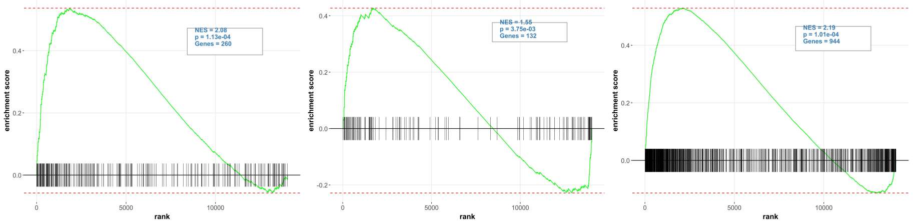

Supplementary Information 9

Bidirectional GSEA validation—enrichment of model group upregulated gene sets in three independent IPF datasets (GSE17978, GSE47460, and GSE213001).

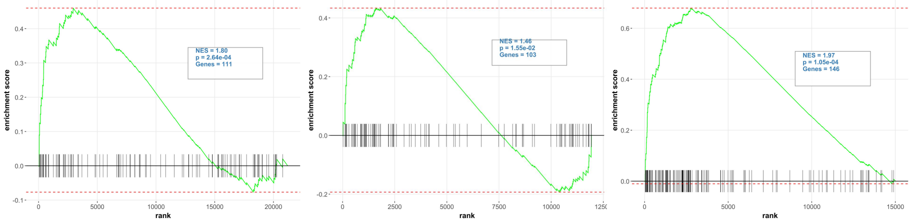

Supplement: Supplementary file 1 [file DataSheet1.zip › Supplementary File/Supplementary Information.pdf]
